# Measuring negative emotions and stress through acoustic correlates in speech: A systematic review

**DOI:** 10.1371/journal.pone.0328833

**Published:** 2025-07-24

**Authors:** Lilien Schewski, Mathew Magimai Doss, Guido Beldi, Sandra Keller

**Affiliations:** 1 Department for Biomedical Research (DBMR), University of Bern, Bern, Switzerland; 2 Department for Visceral Surgery and Medicine, Bern University Hospital, University of Bern, Bern, Switzerland; 3 Graduate School for Health Sciences, University of Bern, Bern, Switzerland; 4 Idiap Research Institute, Martigny, Switzerland; Catholic University of the Sacred Heart: Universita Cattolica del Sacro Cuore, ITALY

## Abstract

Speech analysis offers a non-invasive method for assessing emotional and cognitive states through acoustic correlates, including spectral, prosodic, and voice quality features. Despite growing interest, research remains inconsistent in identifying reliable acoustic markers, providing limited guidance for researchers and practitioners in the field. This review identifies key acoustic correlates for detecting negative emotions, stress, and cognitive load in speech. A systematic search was conducted across four electronic databases: PubMed, PsycInfo, Web of Science, and Scopus. Peer-reviewed articles reporting studies conducted with healthy adult participants were included. Thirty-eight articles were reviewed, encompassing 39 studies, as one article reported on two studies. Among all features, prosodic features were the most investigated and showed the greatest accuracy in detecting negative emotions, stress, and cognitive load. Specifically, anger was associated with elevated fundamental frequency (F0), increased speech volume, and faster speech rate. Stress was associated with increased F0 and intensity, and reduced speech duration. Cognitive load was linked to increased F0 and intensity, although the results for F0 were overall less clear than those for negative emotions and stress. No consistent acoustic patterns were identified for fear or anxiety. The findings support speech analysis as a useful tool for researchers and practitioners aiming to assess negative emotions, stress, and cognitive load in experimental and field studies.

## Introduction

While spoken communication conveys information through its content, it also reveals the speaker's emotional state through tone and other vocal characteristics. Negative emotions or stress in group or team communication can be contagious, escalate interpersonal tensions, or simply indicate the emotional state or stress level of one or more team members [1]. In an operating room, for example—where teamwork is an essential component of the work—negative emotions and stress can impair both technical performance [2] and nontechnical skills, such as communication (e.g., speaking-up behaviors) and decision-making [3,4].

Human emotions can be understood along two dimensions: valence, which describes how positive or negative an emotion is, and arousal, which reflects the intensity of the emotion [5]. *Negative emotions* are affective states characterized by negative valence and, in many cases, high arousal—such as anxiety, anger, and frustration. These emotional states are often accompanied by physiological activation and can trigger stress responses. *Stress* refers to the physiological and psychological responses to perceived threats or challenges that are appraised as exceeding an individual's available resources [6]. Stress can intensify the experience of negative emotions, complicating emotion regulation. Further, *cognitive load*—defined as the level of mental effort required to process a given amount of information [7]—can act as a stressor when the cognitive resources available are insufficient. Therefore, although negative emotions, stress, and cognitive load are distinct constructs, they are interrelated and may lead to overlapping acoustic patterns in speech. We thus decided to include the three constructs in the review and present the results in a way that allows disentangling similarities and differences in the acoustic correlates associated with each construct.

There is evidence that internal states, including emotions, stress, and cognitive load, affect how we speak. Speech patterns are influenced by physiological interactions between the central nervous system (CNS), autonomic nervous system (ANS), and the vocal production system [8]. When the ANS is activated in response to stress, it triggers physiological responses such as increased heart rate, changes in respiratory rate, and muscle tension [9], which affect the vocal folds and alters the way we sound [10]. Humans can correctly identify different emotional states in speech; however, human ratings demonstrate limitations, such as inconsistent accuracy in identifying different emotional states [11]. In contrast, speech analysis offers a fast, non-invasive, and unobtrusive alternative by examining specific features of speech, known as acoustic correlates [12]. These correlates can be categorized into three main groups:

*Prosodic features* are elements of speech—such as intonation (the rise and fall of fundamental frequency), energy pattern (loudness), rhythm (the timing of speech), and duration—that relate to long segments of speech, such as sentences, words, syllables, and expressions [13]. These are commonly referred to as suprasegmental features. Typically, they are derived through short-term processing of the speech signal to extract acoustic correlates such as fundamental frequency and short-term energy across different time windows. These correlates are then parameterized at the turn or utterance level (i.e., over long segments of speech). For a detailed description of the acoustic correlates, refer to Table 1.

**Table 1:** Classification of acoustic correlates used in these studies.

Acoustic correlates	Description
**Spectral Features**	
Spectral centroid/Spectral center of gravity in Hz	Center of the gravity of the magnitude spectrum (perceived brightness of a sound) [14].
Normalized skewness	Third order moment of magnitude spectrum, measuring symmetry around centroid [15].
Normalized kurtosis	Fourth order moment of magnitude spectrum measuring flatness or shape of distribution [15].
Twelve Mel-frequency cepstral coefficients (MFCCs)	Short-term spectral-based features; tend to represent the envelope of speech magnitude spectrum in a compact form [16].
Power spectra/spectral energy	Variance of the signal, which is the average squared deviation from the mean Energy [17].
Formant frequencies (F1, F2, F3, F4)	Natural resonance of the vocal tract [18].
Hammarberg Index	Ratio of strongest energy peak in the 0–2000 Hz region to the strongest peak in the 2000–5000 Hz region [19].
Energy balance frequency (EBF) in Hz	Frequency, for which the energies in the lower and upper frequency bands are closest [20].
Spectral tilt (dB/Hz)	Slope of envelope of short-term spectrum of a speech signal; indicates relative distribution of energy across low frequencies and high frequencies [21].
Cumulative spectral probability diagrams (CSPD)	A cumulative histogram of sound level derived from the discrete envelope spectrum [22].
**Prosodic Features**	
Fundamental frequency (F0) in Hz	Rate of vibration of the vocal folds; measured in cycles per second or Hertz [18].
Amplitude/Intensity in dB	Amplitude is related to the energy of a wave. The more energy a wave has, the higher its amplitude. Intensity measures the amount of energy transmitted by a wave per unit area. For sound waves, intensity is proportional to the square of the amplitude [23].
Sound pressure level in dB	Pressure variation in a sound wave relative to a reference value. Indicates, how loud a sound is [24].
Energy in dB	Energy of a speech signal, expressed in dB.
Time/Duration of speech	The length of sounds in time units ms/s.
Articulation rate/ Speech rate/ Words-per-Minute (WPM)	Articulation rate = Pace at which speech segments are produced, without taking into account pauses. Speech rate includes pause intervals [25]. Words-per-Minute (WPM) represents the number of words spoken in one minute.
Duration/Number of pauses	Frequency and duration of pauses during speech.
Syllable length Syllables per second	Syllable length = Duration/time to pronounce a single syllable, including its voiced and unvoiced segments [26]. Number of syllables per second (total number of syllables divided by the time to pronounce them) [27].
**Voice Quality Features**	
Harmonics-to-noise ratio (HNR) in dB	Ratio of harmonic (periodic) sound to inharmonic (noise) sound in a voice signal [28]. Low HNR values indicate higher level of spectral noise and more breathy voice quality.
Low-to-high spectral energy ratio (L/H ratio)	Balance between low and high frequency energy sounds in a voice signal. Here it is the ratio of spectral energy below and above 4000 Hz [29].
Cepstral peak prominence (CPP)	Relative amplitude of the cepstral peak [30].
Jitter/Frequency perturbation (PPQ)	Cycle-to-cycle variations in glottal pulse timing during voicing [28].
Shimmer/Amplitude perturbation (APQ)	Cycle-to-cycle variations in glottal pulse amplitude during voicing [28].
N	Number of periods in vowel signals after segmentation. Indicator of vowel duration [31].
Digital amplitude length (DAL)	Estimate of the Euclidean length of a signal, obtained by computing the sum of absolute amplitude differences between successive samples. When the summation is computed between two glottal pulses, DAL_T0_ represents the estimate of pitch period length [31].
High-frequency harmonic energy (SPI)	The ratio of the lower frequency harmonic energy in the range 70–1600 Hz to the upper-frequency harmonic energy in the range 1600–4500 Hz. SPI represents the harmonic structure of the spectrum [32].
Voice turbulence Index (VTI)	Average ratio of the spectral inharmonic high frequency energy in the range 2800–5800 Hz to the spectral harmonic energy in the range 70–4500 Hz. VTI measures the relative energy level of high frequency noise [32].

This table categorizes and describes the acoustic correlates measured in the included studies. The features are grouped into Spectral Features, Prosodic Features, and Voice Quality Features, with each category representing different acoustic correlates used. Abbreviations: *MFCC = Mel-frequency cepstral coefficients; F0 = Fundamental frequency; HNR = Harmonics-to-noise ratio; PPQ = Pitch perturbation quotient; APQ = Amplitude perturbation quotient; DAL = Digital amplitude length; SPI = Soft phonation index; VTI = Voice turbulence index; L/H ratio = Low-to-high spectral energy ratio; CPP = Cepstral peak prominence; F1, F2, F3, F4 = Formant frequencies 1, 2, 3, and 4; EBF = Energy balance frequency; CSPD = Cumulative spectral probability diagrams; dB = Decibels; Hz = Hertz; ms = Milliseconds; WPM = Words per minute.*

This table summarizes the key characteristics of the studies included in the systematic review, including study design, the emotion or state being measured, the task or source used to elicit emotions or stress, speech measures analyzed, study results, and quality assessment measures. Abbreviations are defined in Table 1; *n.i.* *=* *no information given in the study.*

*Voice quality features* describe attributes of a voice's sound, such as breathiness and smoothness. Common features include jitter (small variations in pitch), shimmer (small variations in loudness), and the harmonics-to-noise ratio (HNR), which indicates how clear a voice sounds. Changes in these features can signal vocal strain or indicate certain disorders [33,34].

*Spectral features* relate to the frequency content of the sound signal. A common feature is the Mel Frequency Cepstral Coefficient (MFCC), which captures characteristics of speech sounds and is widely used in speech processing (e.g., speaker recognition) [35].

These acoustic correlates map different emotions, as different emotions share distinct acoustic profiles in speech [36,37]. Emotions with the same level of valence and arousal share similar acoustic profiles [38]. Thus, we expect similarities between the acoustic profiles of negative emotions, stress, and cognitive load. However, the current state of research on identifying accurate markers of these states in speech is marked by inconsistency and variability across studies. As a result, a consensus on the acoustic correlates of negative emotions, stress, and cognitive load in speech has yet to be established.

The objective of this systematic review is to identify key features of speech that indicate negative emotions, stress, and cognitive load. By establishing a comprehensive overview of the current state of research, this review contributes to a deeper understanding of how these states are externalized through speech. By doing so, we aim to provide guidance for practitioners and researchers by offering recommendations for measuring affective states in communication.

The question the present literature review aims to answer is*: What are acoustic correlates of negative emotions, stress, and cognitive load in speech in healthy adults?*

## Methods

This systematic search is reported according to the PRISMA 2020 guidelines [39]. A protocol for this review was published in PROSPERO (CRD42024525922).

### Eligibility criteria

Studies were included if they met the following criteria: (1) they were original, peer-reviewed journal articles; (2) they reported experiments or field studies; (3) they examined the acoustic correlates of negative emotions, stress or cognitive load in speech; and (4) they involved healthy adult participants.

Studies published from the inception of the databases until March 19, 2024, were included.

We excluded studies that focused on participants with (1) disabilities; (2) psychological disorders (e.g., schizophrenia, depression, and autism); (3) neurodegenerative disorders (e.g., Alzheimer’s disease, dementia, Huntington’s disease, Parkinson’s disease); or (4) speech disorders (e.g., aphasia, dysphonia). Studies conducted with children or animals were excluded.

Additionally, we excluded review articles and studies that (1) focused solely on methods for feature extraction for machine learning models, (2) were related to speaker or language identification, or (3) were conducted with actors or simulated emotions.

### Search strategy

A systematic search was conducted in March 2024 across four electronic databases: PubMed, PsycInfo, Web of Science, and Scopus. The search strategy included terms related to *acoustic correlates*, *tension* (e.g., stress, negative emotion, cognitive load, frustration, negative affect, anger, aggression), and *speech* (e.g., oral communication, voice communication). For a detailed description of the search string, refer to S1 Appendix in the Supporting Information.

Two independent reviewers (LS, SK) performed the selection of articles for inclusion using Rayyan [40]. Studies were selected based on their title, keywords, and abstract. The reviewers were blinded to each other's decisions; the concordance rate was 97% for abstract screening and disagreements were resolved by discussion.

#### Data extraction.

The extracted data included the following information: author(s), year of publication, study type, country of publication or study location, measured emotion or stress, measured acoustic correlates, type of measurement (human vs. automated rating), participants’ demographics (including gender, age, and language), and reported acoustic correlates.

In addition, we extracted information regarding the setting in which emotions were assessed, additional measurement methods employed to validate the emotion, and the presence of a control condition or group. For detailed information on the extracted data, refer to S1 Table in the Supporting Information.

#### Risk of bias assessment.

The methodological quality of the included studies was assessed using the latest version of the Mixed Methods Appraisal Tool (MMAT) [41]. The MMAT is designed for the quality appraisal of empirical studies performed based on a variety of different methodologies: qualitative research, randomized controlled trials, non-randomized studies, quantitative descriptive studies, and mixed methods studies. It is widely used in systematic reviews including mixed methods studies [42,43]. Studies were rated based on five subquestions, with each subquestion answered positively contributing to 20% of the total quality criteria. If a subquestion is rated as only partly positively answered, the study meets 10% of the quality criteria. The overall quality assessment is the sum of the percentages from all sub-questions. Therefore, each study can achieve between 0% and 100% of the quality criteria. In cases where data are missing, studies receive lower scores in the quality assessment.

#### Data synthesis.

For data synthesis, study characteristics were systematically extracted and tabulated in an Excel sheet. The following variables were recorded: author(s), year of publication, study location, study design (field study or experiment), emotion or stress measured, and acoustic correlates assessed. Additionally, details were recorded on the task or source used to assess or elicit negative emotions or stress, the type of speech samples analyzed (e.g., sentences, syllables, vowels), the main findings of each study, and the quality assessment scores.

To synthetize the findings, we grouped the results into three distinct categories:

1. Stress-related
2. Negative emotion related
3. Cognitive load-related

Within each category, studies were further classified according to the type of acoustic features analyzed, grouping them into spectral, quality, and prosodic features. These categories were defined based on common acoustic parameters used in the included studies. The findings are summarized descriptively, with particular focus on the key patterns observed in relation to the specific psychological state (negative emotion, stress, or cognitive load) being assessed. Differences in study outcomes were noted and discussed in relation to variations in study design and measurement techniques.

## Results

### Risk of bias assessment

The Mixed Methods Appraisal Tool (MMAT) was used to assess the quality of the studies included. The results of this assessment are shown in Table 2. Out of the 39 studies, 35.9% showed a high methodological and reporting quality (scoring >80%), 33.3% showed moderate quality (scoring between 60–79%), and the remaining 30.8% were of low quality (scoring less than 60%). The average MMAT quality score across the 39 studies was 65.4%, indicating moderate overall quality. The most common reasons for low and moderate scores in non-randomized quantitative studies were unclear or missing information about the methodology, particularly regarding inclusion and exclusion criteria and participant recruitment methods. Other contributing factors included the lack of consideration for confounding variables such as smoking, caffeine, and alcohol, and the absence of additional measures (e.g., physiological or subjective measures) or sufficient control conditions. For a detailed overview, refer to S2 Table of the Supporting Information.

**Table 2:** Characteristics of the studies included in the systematic review (N = 38).

Study ID	Author(s), publication year	Origin of study	Study Design	Emotion or stress being measured	Task/Source of negative emotion or stress	Tool used to measure acoustic correlates	What kind of speech is being measured?	Acoustic correlates	Results	Quality Assessment MMAT (% quality criteria met)
1	Abur et al. (2023)	Boston, USA	Experiment	Cognitive Load	Stroop task (congruent/in-congruent condition)	PRAAT	Read-aloud sentences	CPP L/H ratio F0 Sound pressure level	No statistically significant results.	70%
2	Alvear et al. (2012)	Malaga, Spain	Experiment	Stress	Mental arithmetic task	Multi-Dimensional Voice Program (MDVP) Model 5105, KayPENTAX™	Spanish Vowel/æ/	Mean F0	Mean F0 increases.	80%
3	Biassoni et al. (2016)	Milan, Italy	Experiment	Hot Anger, Cold Anger	Anger provoking simulated driving scenario	Multi-Speech Analysis Workstation version 2.5.2 (Kay Pentax)	Spontaneous speech	F0 (mean, std, min, max) Energy (mean, std, min, max) Time (vocal string length, speech length, number and duration of pauses)	Increase in F0 min range Increase in max Energy No other changes in F0 and energy No changes in time parameters.	70%
4	Bonner (1943)	South Carolina, USA	Experiment	Fear/ Tension	Holding oral presentations	n.i.	Speech of presentations	F0 mean, range of F0 Rhythm (Hypha-time, pause-time and total time)	No constant trend. Increased F0 mean Wider F0 range Hypha (syllable)-length increase Longer pause-time.	50%
5	Boyer et al. (2018)	Toulouse, France	Experiment	Cognitive Load	Memory task (recall)	PRAAT	Vowels	Mean F0, Fmod, Jitters (J1, J2, RAP, PPQ5, DDP) Shimmers (S1, S2, APQ3, APQ5, APQ11, DDA) N, DAL, DALT0, DALT0/T0, jittDALT0, jittDALT0/T0 Mean HNR, SDHNR F1, F2, F3, F4 Spectral center of gravity Normalized skewness Normalized kurtosis Twelve MFCCs Three energy differences between two bands of the vowel’s fast Fourier transform spectrum EBF (in Hz) Spectral tilt (dB/Hz)	Mean F0 and SDF0 increase significantly Shimmer 1 and FMod decrease significantly No effect on other shimmers No effect on jitters N increases significantly DALT0 decreases significantly No significant changes in DALT0/T0. JittDal0/T0 and jitDal/T0 decrease significantly HNR varies significantly No other spectral parameters vary significantly.	90%
6	Brenner et al. (1994)	Washington DC, USA	Experiment	Cognitive Load	Manual tracking task (Workload Demand)	A speech analysis program was developed (no further information given)	Counting of numbers	Mean F0 Speaking rate (syllables per second) Vocal intensity (loudness) Vocal jitter Vocal shimmer Derived speech measure (combines properties of several speech measures)	Mean F0 increases Increase in loudness (intensity) Increase in speaking rate No effect on vocal jitter and vocal shimmer Derived speech measure increases significantly, especially when jitter was excluded (higher strength).	50%
7	Bucharan et al. (2014)	Missouri, USA	Experiment	Stress	Trier social stress test (TSST) as a public-speaking task and a mental arithmetic task	Calculations	Speech	Total number of WPM Number of pauses Duration of pauses	Number of pauses and duration of pauses increase Total number of WPM not associated with physiological indices of stress.	80%
8	Bulling et al. (2020)	Zurich, Switzerland	Experiment	Stress	Trier social stress test (TSST) as a job interview task and a mental arithmetic task	PRAAT	Speech	Mean F0	Mean F0 increases.	70%
9	Congleton et al. (1997)	Texas, USA	Experiment	Cognitive Load	Simulation of an Airborne Warning and Control System (AWACS) mission scenario	Automated pitch extraction program SWIFFT; it used a peak-to-peak scoring algorithm	n.i.	F0 Jitter Shimmer	F0 increases significantly Less consistent inverse relationship between jitter and cognitive load No changes in shimmer.	50%
10	Fuller et al. (1992)	Colorado, USA	Field Study	Anxiety	Graduate comprehensive examination	Accelerometer (for jitter and shimmer) and computer programs	/a/ and/i/ vowels	Mean F0 Jitter Tenseness (formant frequencies F2, F5)	Lack of validity for mean F0 changes F2 changes significantly, mixed results for other tenseness measures Valid jitter changes.	70%
11	Griffin & Williams (1987)	Florida, USA	Experiment	Stress	Psychomotor and dichotic listening tasks representing the flight environment	Kay Elemetrics (Model 6087) Visi-Pitch; a portable analog instrument that extracts and displays selected vocal parameters in real-time	Utterances	F0 Intensity Word duration in ms	F0 increases significantly Intensity increases significantly Word duration decreases significantly (rapid speech).	40%
12	Hall et al. (2021)	Wales, UK	Experiment (Simulation)	Stress	Simulation of an in situ operating room environment	long-term pitch analysis with PRAAT (autocorrelation method)	Speech	Mean F0 F1, F2, F3, F4	Mean F0 increases significantly No changes in F1, F2, F3, F4.	40%
13	Hecker et al. (1968)	Massachusetts, USA	Experiment	Stress	Arithmetic task under time pressure	Human Rating & Spectrograms	Speech	Mean F0 F0 contour during an utterance Amplitude of glottal pulses (level) Shape or frequency spectrum of each pulse Regularity in shape of successive pulses Initiation of glottal vibration Duration of phonetic segments Precision of articulatory targets for vowels	No homogenous trend, increases and decreases in the acoustic correlates.	40%
14	Hodgins et al. (2010)	New York, USA	Experiment	Threat Response	Structured stressful interview and giving speeches with moderate high social threat	Amadeus Software (https://www.hairersoft.com/)	Speech	Mean F0	Mean F0 increases in perception of controllability.	70%
15	Huttunen et al. (2011a)	Oulo, Finland	Experiment	Cognitive Load	Simulated combat flights	Extensible Markup Language (XML) file with information on timing and pauses; calculation of articulation rate, PRAAT software	Utterances	Articulation rate (Syllabes per second) Mean F1 and F2 front vowel Mean F1 and F2 back vowel	Articulation rate decreases significantly Mean F1 and F2 back vowel increase significantly Increase in mean F1 front vowel, decrease in mean F2 front vowel.	60%
16	Huttunen et al. (2011b)	Oulo, Finland	Experiment	Cognitive Load	Simulated military flights	PRAAT Software; Automated cepstrum-based voiced/unvoiced segmentation and time domain F0 extraction algorithm	Utterances	Mean F0 (utterance level) Mean vocal Intensity	Mean F0 increases Mean vocal intensity increases.	80%
17	Kandsberger et al. (2016)	St. Andrews, UK	Experiment	Emotional Stress	Verona Coding Definitions of Emotional Sequences (VR-CoDES) cues and concerns vs neutral statements from follow-up consultations	PRAAT 5.3.51	Utterances	Mean F0	Mean F0 increases.	80%
18	Kappen et al. (2022)	Ghent, Belgium	Experiment	Psychosocial Stress	Psychosocial stress induction (cognitive task) with negative evaluation	OpenSmile 2.3.0 with the GeMAPS configuration (features were computed locally)	Read-out-loud text	F0 HNR Shimmer Jitter Speech rate	F0 increases HNR increases significantly Shimmer decreases No effect in jitter No effect on speech rate.	80%
19	Kappen et al. (2024)	Ghent, Belgium	Experiment	Acute psychosocial Stress	Psychosocial stress induction (cognitive task) with two stress paradigms (MIST & Cyberball)	OpenSmile 2.3.0 with the GeMAPS configuration (features were computed using Python 3.9.6)	Sentences	F0 Jitter Shimmer HNR Speech rate Voiced segment length	MIST stress paradigm: Significant increase in F0 Significant increase in voiced segments per second (speech rate) and in voiced segment length Significant decrease in Jitter Cyberball: No significant increases/changes No significant change in HNR and shimmer.	80%
20	Lebedeva & Shved (2022)	Moscow, Russia	Experiment	Anxiety	14-day isolation and crowding during a space simulation	PRAAT	Recorded audio reports on mood, well-being and daily activities	F0 Intensity Number of vocal impulses Pause duration (unvoiced segments) Jitter Shimmer	Lower intensity Longer speech pauses Increased shimmer No other results have been reported.	40%
21	Lee & Redford (2015)	Oregon, USA	Experiment	Cognitive Load	Complex span task remembering sequence of letters or spatial locations (verbal or spatial working memory)	PRAAT	Sentences	F0 range Normalized measure of F0 variation Mean normalized sequential variability in vowel durations (nPVI) Error rates Prosodic breaks Articulation rate	Error rate increases Faster speech Fewer prosodic breaks No effect on spoken rhythm & nPVI No effect on F0 correlates.	50%
22	Li et al. (2023)	Chengdu, China	Experiment	Anxiety	Presentation in English for non-English major students	ADPCM algorithm & human raters	Speech (oral presentation)	Mean F0 Short-term energy (STE) Formant Frequency (FM/F1) MFCC1-MFCC12 Brightness (power in speech signals)	Mean F0 MFCC1std FMstd STEstd All correlated with self-reported anxiety.	60%
23	Lively et al. (1993)	Indiana, USA	Experiment	Cognitive Load	Compensatory visual tracking task with increased difficulty	Digital signal processing techniques; Linear predictive coding (LPC) to calculate short-term-spectrum	/h/-vowel-/d/ Utterances embedded in the sentence frame “Say hVd again”	Amplitude (Intensity) Amplitude variability Spectral tilt F0 variability F0Std Duration (phrase duration) Formant frequencies	Increased amplitude (Intensity) Sign. Increase in amplitude variability from one utterance to the next in 4 out of 5 speakers Spectral tilt decreases, without associated change in amplitude No consistent effect on F0 variability Significant decrease in F0 Std Significant shorter phrase duration No significant change in Formant Frequencies.	40%
24	MacPherson et al. (2017)	Boston, USA	Experiment	Cognitive Load	Stroop task (congruent/ incongruent condition)	MATLAB program	repeated read-aloud sentences	F0 Sound pressure level Cepstral peak prominence (CPP) L/H ratio	Increased CPP Lower L/H ratio No change in F0 or sound pressure level.	90%
25	Mendoza & Carballo (1998)	Granada, Spain	Experiment	Cognitive Load	Different cognitive tasks (tongue twister, tongue twister with delayed auditory feedback, reading and inverse alphabet reading) in a stressful experimental environment (time-pressure, fear of getting a bad grading)	Multi-dimensional Voice Program (MDVP) by Kay Elemetrics Corp.	Prolongation of the vowel/a/	F0, F0 range, F0 (STD) Jitter Shimmer Noise-to-Harmonic Ratio (NHR) High-frequency harmonic energy (SPI) Voice Turbulence Index (VTI)	F0 increases Decreased jitter Decreased shimmer Increased SPI Decreased VTI No other changes.	50%
26	Pisanski & Sorokowski (2021)	Wroclaw, Poland	Field Study	Stress	Real-life oral examination situation	PRAAT & Human Ratings	read-aloud sentences	F0, F0 mean, F0 min, F0 max, F0SD F1-F4 Formant spacing (DeltaF) HNR Jitter Shimmer Speed of speech (duration and words per minute WPM)	F0 increased Greater DeltaF Increased speed of speech (more WPM, shorter duration) No other changes.	60%
27	Pisanski et al. (2016)	Wroclaw, Poland	Field Study	Stress	Real-life oral examination situation	PRAAT	spontaneous speech and read-aloud sentences	Mean F0 F0 min F0 max F0SD	F0 mean increased significantly F0 min increased significantly No sign. Changes in F0SD and F0 max.	80%
28	Pisanski et al. (2018)	Wroclaw, Poland	Experiment	Psychosocial Stress	Trier Social Stress Test (TSST) containing an interview-style oral presentation and social evaluation	PRAAT	free speech (baseline) and speech during TSST (interview speech during task)	Mean F0, F0 range, F0 variation Jitter Shimmer HNR	Mean F0, F0 range and F0 variation change significantly No other significant changes.	90%
29	Rochman et al. (2008)	Beer Sheva, Israel	Experiment	Unresolved Anger	Mood-induction procedure (study 1)	PRAAT 4.1.2 and MDVP (Mutli Dimensional Voice Program)	Utterances	Mean F0 F0 range Amplitude range F0 perturbation (PPQ) Amplitude perturbation (APQ) Articulation rate (WPM)	Mean F0 increased significantly F0 range increased significantly WPM increases significantly Amplitude range increases significantly No increase in APQ and PPQ.	80%
30	Ruiz et al. (1996)	Toulouse, France	1) Experiment 2) Field Study	Stress	1) Stroop task (congruent/ incongruent condition) 2) Cockpit voice recording of a crashed airplane	pitch tracking algorithm implemented on 5500 Kay analyzers & CSL 4300 ILS software cepstrum-based measurements comparative observations of sonograms for spectrum changes estimation of cumulative spectra probability diagram (CSPD) Formant Frequencies with 5500 Kay DSP analyzer	Vowels and Utterances	Mean F0 µ index Δ area Cumulative Spectral Probability Diagrams (CSPD) Spectral balance frequency Fs F1, F2, F3 Distances to the F1-F2-F3 space center	1) Mean F0 increases significantly µ index increases Significant variations in F1,F2,F3 No relation with Δ area variations No effect on F1-F2-F3 space center. 2) Mean F0 increases significantly.	1) 30% 2) 90%
31	Sabo & Rajcani (2017)	Bratislava, Slovakia	Experiment	Stress	Communication task via a computer game called “dismantling a bomb”	n.i.	Speech (interactions)	Mean F0 Mean Intensity	Mean F0 increases significantly in 4 out of 5 speakers Speech intensity increases.	50%
32	Sobin & Alpert (1999)	New York, USA	Experiment	1) Fear 2) Anger	Emotion induction procedure by reading stories that elicit negative emotions	Human ratings	Prototype sentences	F0 (Mean), F0 variance Volume, volume variance Speaking rate Duration of speech Duration of pauses Number of pauses	Fear: Increased F0 (Mean), F0 variance, volume, speaking rate. Decreased duration of speech, duration of pauses, and number of pauses. Anger: Increased F0 variance, volume, volume variance, speaking rate. Decreased F0 (Mean), speech duration, duration of pauses and number of pauses.	60%
33	Sondhi et al. (2015)	Haryana, India	Field Study	Stress	Audio clips from FM broadcast in real-life stressful conversations where the host provokes the subjects	PRAAT software 5.356	Whole utterances	Mean F0 F1, F2, F3, F4	Mean F0 increases F1, F2, F3, F4 decrease.	70%
34	Streeter et al. (1983)	New Jersey, USA	Field Study	Stress	Tape recordings of telephone conversation of the system operator and his superior system operator before the New York blackout	Linear predictive coding analysis & human listener ratings	Utterances	Mean F0, F0 max, F0 min, F0std, Mean Amplitude, SD Amplitude, Max Amplitude Number of words/s	No trend observable in speech analysis Listeners perceive increased F0 amplitude levels and increased variability in F0 and amplitude levels as stressful.	100%
35	Tavi (2017)	Joensuu, Finland	Field Study	Stress	Authentic recorded calls to emergency services	PRAAT and ProsodyPro for jitter, shimmer and HNR	high front/i/-Vowels	Median F0 Jitter Shimmer Harmonics to noise ratio (HNR) Hammarberg Index Duration of/i/-vowels F1, F2, F3 Dispersion between F1-F2	Higher F0 (Median) NLower shimmer No changes in jitter Lower HNR Lower Hammarberg index No difference in duration of/i/-vowels under stress Higher F1 Lower F2 and F3 Narrower Formant dispersion.	90%
36	Taylor et al. (2016)	Indiana, USA	Experiment	Stress	Social stressors in group settings	sociometric badges	group interactions/ discussions (real speech)	F0Std	Greater deviation of F0std with F0 in social stress task (significant) lower Greater deviation of F0std with F0 in problem-solving task higher (marginally significant).	60%
37	Tolkmitt & Scherer (1986)	Giessen, Germany	Experiment	1) Cognitive Load 2) (Emotional) Stress	Slide presentations showing 1) logical problems or 2) human bodies with diseases or injuries	Analogue Extraction (e.g., LPC method)	Utterances, sentences, and vowels	F0 mean, F0 floor F1, F2 power spectra/ spectral energy Formant Distances	No significant consistent changes in F0 (mean) across conditions. Changes vary depending on sex and personality coping style (anxiety denying vs. high anxious). Anxiety denying females showed significant changes in power spectra/spectral energy and Formant Distances. F0 floor as a more sensitive marker than F0 mean for stress.	60%
38	Wittels et al. (2002)	Vienna, Austria	Field Study	(psycho- emotional) stress	Real-life condition of military training task	Speech analysis algorithm by Lüdge & Gips (1986)	Counting from 1–10	F0 mode	F0 mode increased significantly.	70%

**Fig 1:**
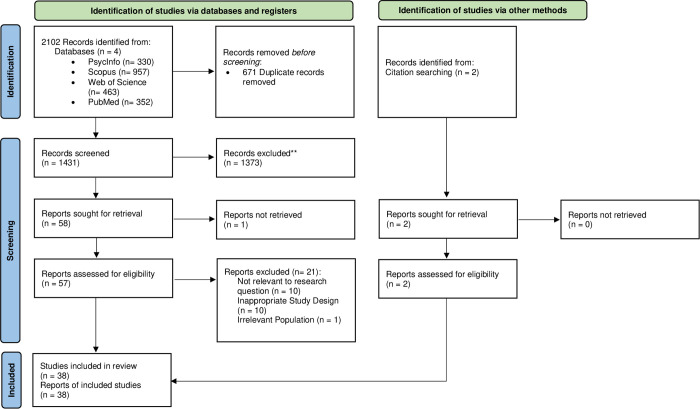
PRISMA 2020 flow diagram for new systematic reviews. Adapted from Page et al. [39].

Fig 1 illustrates the results of the search strategy. After the removal of duplicates, screening of abstracts and titles, full-text screening, and risk of bias analysis, 36 articles were included for data extraction (as detailed in Table 1). Additionally, after reviewing the reference lists of relevant studies, two additional articles were identified and included, bringing the total number of studies to 38. One article, a conference proceeding by Lee & Redford [44], was identified in the systematic search as a peer-reviewed work and, after discussion among the authors, met the inclusion criteria.

### Study characteristics

Among the 38 articles included, 78.9% *(n* *=* *30)* employed an experimental design, whereas 18.4% *(n* *=* *7)* were field studies conducted in areas such as aerospace, healthcare, broadcasting, and academic contexts. One of the articles [22] reported both a field study and a laboratory experimental design, bringing the total number of studies to *n* *=* *39*.

Most studies were conducted in the USA *(n* *=* *17)*, followed by the UK (*n* *=* *3)*, Finland *(n* *=* *3)*, Belgium *(n* *=* *3)*, and other European and Asian countries. The majority of studies targeted stress (*n* *=* *20*) and cognitive load (*n* *=* *10)*. Studies on negative emotions (*n* *=* *9)* focused on anger (*n* *=* *3),* anxiety (*n* *=* *3*), fear/tension (*n* *=* *2*), and threat response (*n* *=* *1*).

Most studies *(n* *=* *10)* elicited stress or cognitive load using cognitive tasks, such as the Stroop Task and arithmetic tasks performed by participants under time pressure. Eight studies used simulated scenarios in contexts like airforce, military, operating rooms, and driving scenarios. Additional contexts included: 1) passive mood induction procedures, such as follow-up consultations and viewing slide presentations or images eliciting negative emotions *(n* *=* *4)*; 2) audio recordings, including calls to emergency services, recordings of real-life stressful conversations, and broadcast recordings *(n* *=* *4)*; 3) social stressors, such as Trier Social Stress Task (TSST), Cyberball, and cognitive tasks with negative evaluations *(n* *=* *6)*; 4) oral examinations *(n* *=* *3),* and delivering presentations or speeches *(n* *=* *2).*

In addition to acoustic analyses of negative emotions, stress, and cognitive load, some studies (*n* *=* *20*) used physiological or subjective measures to validate the presence of these states. These measures include heart rate (*n* *=* *7*), blood pressure (*n* *=* *1*), pulse rate (*n* *=* *3*), self-reports (*n* *=* *6*), skin conductance (*n* *=* *4*), palmar sweating (*n* *=* *1*) behavioral signals (*n* *=* *3*), cortisol levels (saliva) (*n* *=* *5*), and pupillary response (*n* *=* *1*). A detailed overview is provided in S3 Table in the Supporting Information.

Commonly used tools or methods to analyze the acoustic features were PRAAT, Multi-speech software tools from KayPENTAX™, the open-source toolkit OpenSmile, and human ratings. Regarding measured speech sequences, most studies focused their analyses on natural speech, read-aloud sentences, utterances, and vowels. A few studies also examined counting and speech produced during presentations. For further details, see Table 2.

All 39 studies examined prosodic features, with 14 also investigating voice quality features and 12 analyzing spectral features. Some studies examined multiple feature categories.

Prosodic features were examined 69 times, with 52 instances showing a correlation or relationship with negative emotions, stress, or cognitive load. Voice quality features were examined 37 times, detecting these states in only 18 cases. Of the 19 instances where spectral features were examined, 8 showed at least one relationship with negative emotions, stress, or cognitive load. Thus, compared to spectral and voice quality features, prosodic features showed overall better accuracy in detecting negative emotions, stress, and cognitive load.

The following section presents the findings on acoustic correlates associated with negative emotions, stress, and cognitive load.

### Negative emotions

Within the category of negative emotions, the studies investigated anxiety, anger, fear, and responses to perceived threat. The acoustic patterns associated with each are presented below.

**Anxiety:** In the category of spectral features, two studies reported changes in formant frequencies related to anxiety. Li and colleagues reported changes in the first formant (F1) and in the standard deviation of MFCC1 [45], while Fuller and colleagues reported changes in the second formant (F2) [46].

For prosodic features, findings on fundamental frequency (F0) were mixed. One study reported a correlation between F0 and self-reported anxiety [45], whereas two other studies found no significant changes [46,47]. Anxiety was also associated with decreased intensity and increased pause duration [47] and short-term energy (STE) [45].

**Fig 2:**
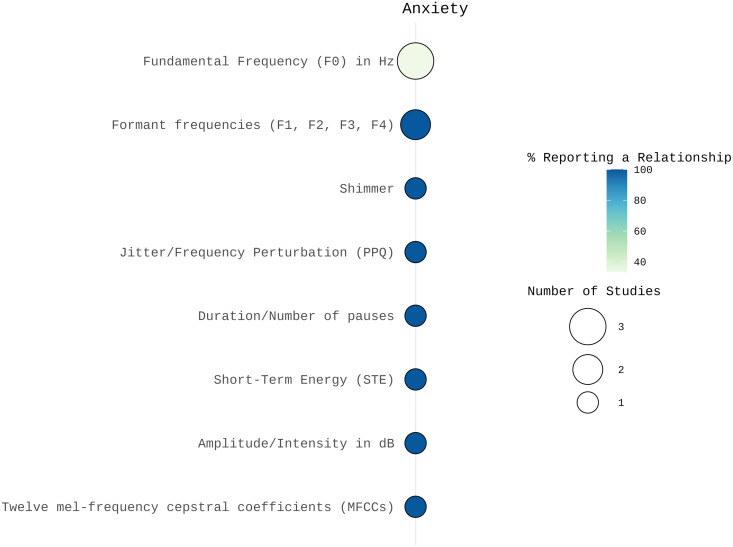
Number of studies and percentage of studies reporting a relationship between acoustic features and anxiety visualized using SRplot [48]. The size of the balloons indicates the number of studies examining each acoustic correlate. The color represents the percentage of studies reporting a relationship, with darker colors indicating a higher proportion of findings supporting an association between the acoustic feature and anxiety.

Both studies examining the voice quality features jitter or shimmer reported associations with anxiety [46,47]. Fig 2 illustrates the percentage of studies reporting relationships between the acoustic features and anxiety, with larger bubbles indicating a greater number of studies.

**Anger:** In the category of prosodic features, anger was associated with increased mean F0 and related features in two studies [49,50], whereas Sobin and Alpert [51] reported a decrease in mean F0 but an increase in F0 variance. Anger was also linked to higher volume [51] and greater maximum energy levels [49].

**Fig 3:**
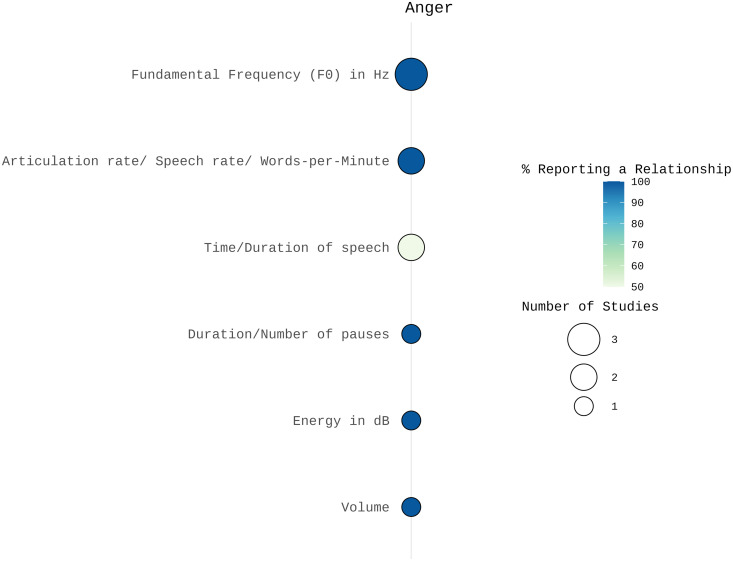
Number of studies and percentage of studies reporting a relationship between acoustic features and anger visualized using SRplot [48]. The size of the balloons indicates the number of studies examining each acoustic correlate. The color represents the percentage of studies reporting a relationship, with darker colors indicating a higher proportion of findings supporting an association between the acoustic feature and anger.

Results on temporal features were mixed. Sobin and Alpert [51] reported fewer and shorter pauses and reduced speech time, while Biassoni et al. [49] found no changes. Speech rate consistently increased for anger across two studies [50,51], whereas the voice quality features jitter and shimmer showed no association with anger [50]. Refer to Fig 3 for a visual summary of the results.

**Fig 4:**
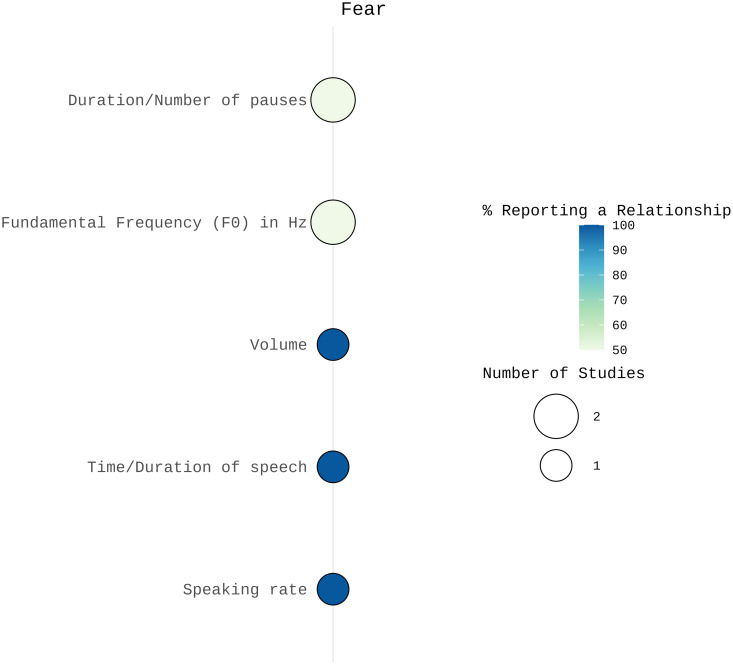
Number of studies and percentage of studies reporting a relationship between acoustic features and fear visualized using SRplot [48]. The size of the balloons indicates the number of studies examining each acoustic correlate. The color represents the percentage of studies reporting a relationship, with darker colors indicating a higher proportion of findings supporting an association between the acoustic feature and fear.

**Fear:** For prosodic features, Sobin and Alpert [51] found that fear was associated with increases in both mean F0 and F0 variance. In addition, they reported a faster speaking rate, shorter speech duration, fewer and shorter pauses, and increased volume. In contrast, Bonner [26] observed changes in F0 and temporal parameters but found no consistent trends in the number and duration of pauses or syllable length. Refer to Fig 4 for a visual summary of the results.

**Threat response:** Threat response was associated with increased mean F0 in one study [52].

### Stress

Spectral Features: Three studies reported significant variations in formant frequencies under stress [22,53,54], while two studies found no significant changes [18,55]. Other spectral features, such as power spectra/spectral energy, and the Hammarberg Index, showed promising results [53,56]; however, the number of studies investigating these features is limited.

Prosodic Features: Several parameters related to fundamental frequency (F0) have been identified as correlates of stress. Notably, fifteen out of nineteen studies reported a significant increase in mean F0 [18,22,52–55,57–65]. However, four studies found no consistent trend or increase in F0 [56,66–68]. For example, Kappen and colleagues [66] observed an increase in F0 within the MIST stress paradigm, but not in the Cyberball paradigm. Streeter and colleagues [67] reported no consistent trend using automated speech analysis, yet human raters perceived an increase in F0 amplitude levels and greater variability in F0. In contrast, Taylor and colleagues [68] observed a decrease in F0 in a social stress experimental setting, and Tolkmitt and Scherer found no consistent significant changes in mean F0 across conditions [56].

Two studies found a significant increase in intensity or amplitude under stress [59,65]; Streeter and colleagues [67] found that human raters also perceived an increased intensity, but did not find a consistent trend using automated speech analysis.

The results regarding time or duration of speech and speech rate are heterogeneous. While some studies found a decrease in speech duration [55,59] another study found no effect [69]. Similarly, Pisanski and Sorokowski [55] observed an increase in speech rate under stress while three other studies [61,67,70] found no effect. Kappen and colleagues [66] found an increase in voiced segments per second and in voiced segment length under stress. Buchanan et al. (2014) reported an increase in the number and duration of pauses.

**Fig 5:**
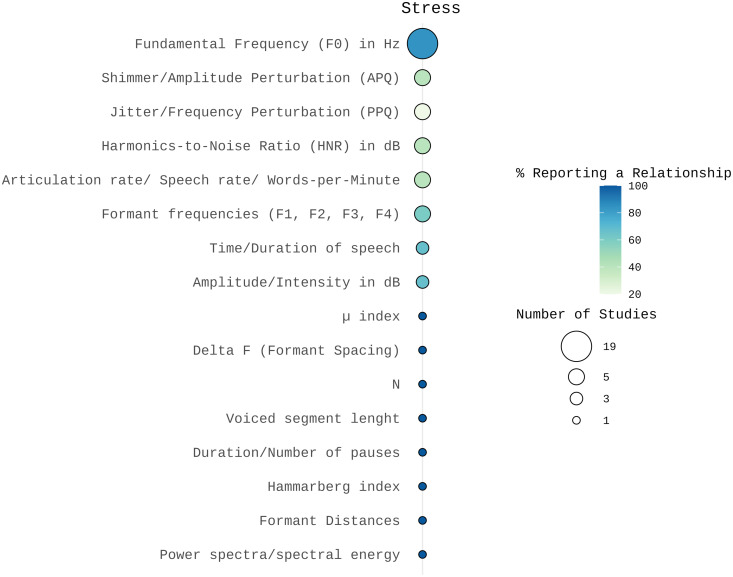
Number of studies and percentage of studies reporting a relationship between acoustic features and stress visualized using SRplot [48]. The size of the balloons indicates the number of studies examining each acoustic correlate. The color represents the percentage of studies reporting a relationship, with darker colors indicating a higher proportion of findings supporting an association between the acoustic feature and stress.

Voice Quality Features: Three studies [55,64,66] found no significant change in Harmonics-to-Noise ratio (HNR) under stress, while two reported significant changes in HNR [47,64] but no consistent trend. While Kappen and colleagues [71] reported an increase in HNR, Tavi [53] reported a decrease in HNR under stress. Results for jitter and shimmer were inconsistent. Whereas two studies reported a decrease in shimmer [53,71] and a decrease in jitter [66], most of the studies found no association between shimmer and stress [53,55,64]. Similarly, the majority of the studies found no association between jitter and stress [53,55,64,71]. However, Kappen and colleagues [66] reported a reduction in jitter for the MIST stress paradigm. Refer to Fig 5 for details.

### Cognitive load

Spectral features: Three studies found no significant changes in formant frequencies [21,31,56]. Huttunen and colleagues [72] reported significant variations in formant frequencies under cognitive load, but the direction of the effect varied across different formants. Regarding other spectral features, a decrease in spectral tilt was observed by Lively and colleagues [21], whereas Boyer and colleagues [31] found no association.

Prosodic features: Most of the studies investigating F0 found a relationship with cognitive load. Six out of ten studies reported an increase in F0 [27,31,73–75], while Lively and colleagues found a decrease in the standard deviation of F0 [21]. Four studies, however, reported inconsistent changes [29,44,56,76].

All three studies investigating intensity or amplitude found an increase under cognitive load [21,27,74].

Findings on speech rate were mixed: Huttunen and colleagues [74] reported a decrease in articulation rate (syllables per second), while Lee and Redford [44] and Brenner and colleagues [27] reported increases in speech and articulation rates. Lively and colleagues [21] reported a decrease in phrase duration, and Lee and Redford [44] reported fewer prosodic breaks.

Voice quality features: Mendoza and Carballo [75] found no change in noise-to-harmonics ratio (NHR) under cognitive load. Regarding the low-to-high spectral energy ratio (L/H ratio), MacPherson and colleagues [76] reported a decrease under cognitive load. Abur and colleagues [29] found a decrease in L/H ratio in older adults but the results were not statistically significant.

**Fig 6:**
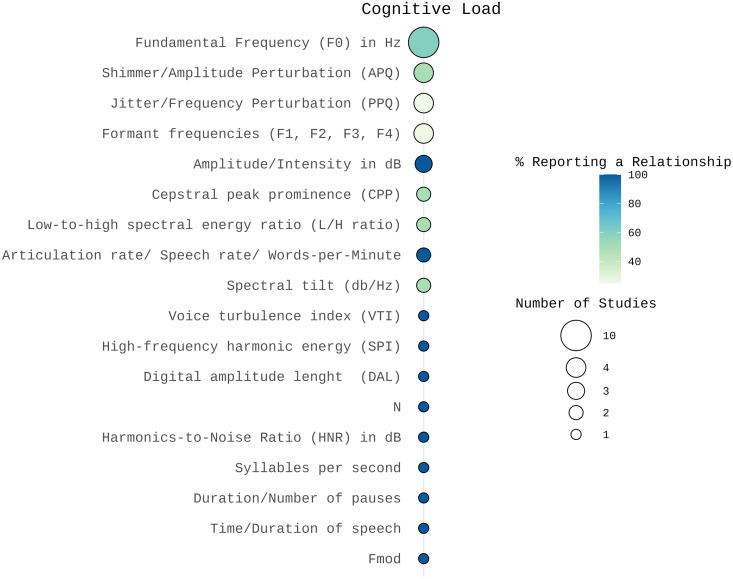
Number of studies and percentage of studies reporting a relationship between acoustic features and cognitive load visualized using SRplot [48]. The size of the balloons indicates the number of studies examining each acoustic correlate. The color represents the percentage of studies reporting a relationship, with darker colors indicating a higher proportion of findings supporting an association between the acoustic feature and cognitive load.

Most studies (n = 3) found no significant changes in jitter and shimmer under cognitive load [27,31,73], although Mendoza and Carballo [75] reported decreases in both features. Additionally, few studies identified an impact of cognitive load on further voice quality features: Mendoza and Carballo [75] reported a decrease in Voice Turbulence Index (VTI) and an increase in high-frequency harmonic energy (SPI), while Boyer and colleagues [31] reported an increase in N and a decrease in DALT0 (see Fig 6 for details).

## Discussion

Our review identified that studies investigated a total of 28 different acoustic correlates in association with negative emotions, stress or cognitive load.

Results for negative emotions showed different patterns for specific negative emotions. Some studies reported changes in formant frequencies and MFCCs associated with anxiety. Findings for F0 were inconsistent, while a few studies found associations with intensity/amplitude, short-term energy, duration or number of pauses, and shimmer and jitter. Anger was associated with increases in F0, speech rate, volume, and intensity but not with Jitter and shimmer. Similarly, fear was associated with faster speech rate and increased volume in one study. No consistent trends were found for F0 or time parameters.

For stress, a majority of studies reported significant increases in mean F0. Similarly, intensity/amplitude increased under stress. Results for speech rate and speech duration were heterogeneous with some studies reporting a decrease in speech duration and an increase in speech rate, while others found no changes. Voice quality features (such as HNR, jitter, and shimmer) showed no consistent trends.

For cognitive load, changes in F0 and intensity/amplitude were reported. However, a few studies found no changes, decreases or no consistent trend for F0. Speech rate findings were mixed, with both increases and decreases reported across studies. Changes in voice quality features were observed in a few studies. Jitter and shimmer showed no consistent patterns.

Across all conditions, increased F0 and increased intensity emerged as good indicators for anger, stress and cognitive load. However, some studies on cognitive load yielded mixed results indicating that F0 may be a slightly less effective marker of cognitive load than of negative emotions and stress. One possible reason is that cognitive load does not directly involve the autonomic nervous system in the same way as negative emotions and stress, leading to none or fewer of the high-intensity physiological responses that affect F0.

Across conditions, speaking rate and voice quality features showed inconsistent results. Interestingly, studies that reported no effect on jitter and shimmer focused on cognitive load, suggesting that further research is needed to explore their association with other emotional states. In addition to these findings, several voice quality features—Voice Turbulence Index (VTI), High-Frequency Harmonic Energy (SPI), N, and Digital Amplitude Length (DAL) – as well as spectral features such as spectral tilt, spectral energy, and the Hammarberg Index, showed a positive association with cognitive load and stress. Nonetheless, currently, studies on these parameters are scarce, limiting our understanding about how they vary depending on different emotions and levels of cognitive load and stress. Specifically, the impact of negative emotions and stress on spectral features remains underexplored, with no studies focusing on how these features may be associated with these states.

The gap in research is compounded by the limited comparability across studies due to differences in experimental settings, variations in stress induction procedures, and challenges in quantifying the type and level of emotion or stress induced [75,77]. It is well known that simulations can elicit the same emotions as real-life situations [78]. In contrast, laboratory studies might cause participants to modify their emotional responses due to increased self-awareness. Furthermore, laboratory-induced stress often results in smaller stress responses compared to real-life situations [79], and certain acoustic correlates might be less effective in detecting subtle changes in negative emotions and stress. Another reason for inconsistency, as suggested in other research, could be the reliance on acted emotional databases, as acoustic variations in spontaneous speech are more subtle than in posed emotional expressions [80]. Moreover, different stress protocols stimulate qualitatively and quantitatively different stress responses [66,81], and thus, inconsistent effects across studies might be explained by differences in stress induction methods [82]. The Trier Social Stress Test (TSST) is a widely recognized tool for inducing psychosocial stress in laboratory settings [83,84], whereas other methods might not evoke a stress response strong enough to elicit observable variation in speech or could induce different stress response patterns. For example, while Kappen and colleagues [66] observed significant changes in acoustic parameters with the MIST stress paradigm (inducing cognitive stress), no significant changes have been found for the Cyberball stress paradigm measuring cognitive load. This difference might be due to the circumstance that the MIST stress paradigm elicits a physiological neuroendocrine stress response while the Cyberball stress paradigm elicits a psychological stress response. In line with this, the direction of the effect can vary with the emotion or stress studied. Taylor et al (2016) found a significantly lower F0 in a social stress task but a marginally significant higher F0 in the problem-solving task. Future research should systematically compare the acoustic features across different types of stressors and report the nature of the stress induction to improve interpretability and cross-study comparability.

It is also important to consider individual differences in stress responses, with the same stressor potentially producing different responses. In line with this, some individuals might not show a physiological stress response and corresponding vocal changes despite being in a stressful condition [55]. Speech changes can result from both involuntary physiological changes and voluntary efforts. Individual coping styles may affect how stress responses are manifested in speech, leading to variations in outcomes. Individual differences in vocal output could be related to the degree of top-down regulation, which is affected by a person’s role, position, and training [8]. Furthermore, individuals may strategically conceal their true emotional states, complicating the measurement of negative emotions and stress in real-life situations. Therefore, some speech patterns might reflect a learned tendency to control the voice rather than a direct effect of autonomic arousal [85]. To minimize inconsistencies across studies, future research should focus on verified emotions and stress states and statistically control for individual differences in stress responses [63]. Using stress induction paradigms that reliably elicit strong stress reactions can also enhance the sensitivity of acoustic analyses to stress-related changes.

A key consideration when interpreting acoustic correlates is the type of measurement used. This review emphasizes associations between acoustic features and self-reports or task-defined conditions, which reflect experienced emotional states, stress, or cognitive load. However, these may differ from physiological indicators or ratings provided by external observers. A significant limitation of this review is the variability in quality of the included studies (overall quality score of 65.4%), with some studies showing poor study design and small sample sizes, limiting the external validity of findings. The critical appraisal indicates that most of the studies reviewed are of moderate quality. This is primarily due to unclear information about the representativeness of participants, the failure to account for potential confounding variables such as smoking, alcohol consumption, and caffeine intake, and the absence of additional control measures. Since we only included studies on healthy adult participants, the findings may not be fully applicable to populations with different characteristics (e.g., age, mental health conditions, or health conditions).

None of the papers in this review included the Teager Energy Operator (TEO) as a feature for emotion recognition. TEO is a nonlinear speech feature used to analyze and classify different emotional states. It is sensitive to the interactions between different frequency components [13]. The advantage of using nonlinear speech features, such as TEO, lies in their ability to detect subtle nonlinear patterns, such as variations in airflow through the vocal tract, which may reflect emotional changes.

While negative emotions, stress, and cognitive load share partially overlapping acoustic profiles – particularly increases in F0 and intensity – this similarity does not preclude differentiation. Instead, it underscores the importance of incorporating multiple acoustic features and integrating them with contextual information to more accurately distinguish between emotional, stressful, and cognitive states. Although single features may be nonspecific, combinations of features can help distinguish between closely related conditions. Therefore, we recommend using multiple features to improve accuracy in identifying these states. Additionally, we suggest incorporating other measurements, such as physiological data or subjective self-reports and assessments. Consequently, this could lead to more precise emotion recognition tools that can enhance real-time detection. This is particularly valuable in complex work environments, where negative emotions and stress can significantly impact teamwork and safety.

To improve future comparability and reduce methodological heterogeneity, the field would benefit from the development and adoption of standardized experimental protocols. These should include clear definitions of the constructs investigated, standardized speech tasks, controlled recording conditions, and consistent inclusion of relevant control variables. Such standardization would enhance transparency, facilitate replication, and support cross-study comparisons. Additionally, the standardized reporting of participant demographics – including age, gender, and language background – and specification of the feature extraction pipeline (e.g., tools used, time window) could improve generalizability across contexts.

## Conclusions

In summary, our systematic review examined the acoustic correlates of negative emotions, stress, and cognitive load in speech. It shows that some, but not all, acoustic features may serve as valid, non-invasive indicators for assessing these constructs. Nonetheless, variability in study design and quality likely contributes to the heterogeneity of results observed in the literature. Despite these differences, F0 and intensity, which are prosodic markers, show strong potential as reliable indicators of emotional arousal, stress, and cognitive load. Future research should focus on these acoustic correlates. Other acoustic correlates, especially spectral features, showed promising results in analyzing stress and cognitive load in speech but require further research. This review also highlights the opportunity to explore whether and how spectral features could serve as markers for negative emotions beyond cognitive load and stress. To date, studies conducted in real-world or workplace settings are scarce, making it difficult to capture the complexity of emotions arising naturally in everyday life. Therefore, more research conducted in real-life settings is needed.

## Supporting information

S1 Appendix Search string and databases. (DOCX)

S1 Table Data Extraction Table. (XLSX)

S2 Table Quality Assessment with the MMAT. (DOCX)

S3 Table Characteristics of the studies included in the systematic review. (DOCX)

S4 Table PRISMA 2020 Checklist. (DOCX)
