# Supplementary material for: Measuring negative emotions and stress through acoustic correlates in speech: A systematic review
Source: PLoS One. 2025 Jul 24;20(7):e0328833. doi: 10.1371/journal.pone.0328833 (PMC12289014; doi:10.1371/journal.pone.0328833)
Supplement: S1 Appendix — (DOCX) [file pone.0328833.s001.docx]

# **S1 Appendix. Search string and databases.**

**Databases used:**

- Scopus (Since 1950)
- Web of Science (Since 1900)
- PubMed (including MEDLINE since 1966)
- PsycInfo (Since 1967)

**Search Strings depending on databases used:**

Following search keywords will be used in each databases:

**Scopus (n= 957 Articles)**

( TITLE-ABS-KEY ( ( acoustic* OR {acoustic cue} OR {acoustic correlate} OR {audio cue} OR {voice stress} OR {acoustic analysis} OR pitch OR {fundamental frequency} OR {sound properties} OR psychoacoustic* OR prosod* ) ) AND TITLE-ABS-KEY ( ( stress OR {cognitive load} OR anger OR frustrat* OR tension OR {negative affect} OR aggress* OR {negative emotion} ) ) AND TITLE-ABS-KEY ( ( speech OR {oral communication} OR {voice communication} ) AND NOT ( animal OR child* OR schizophrenia OR depress* OR autism OR dementia OR Alzheimer OR Huntington OR Parkinson OR aphasia OR dysphonia OR impairment OR damage OR disorder OR disabilit* OR {machine learning} OR { lexical stress} OR {linguistic stress} OR {word stress} OR music OR {compound stress} OR {glottal stop} OR singing OR trans OR {cochlear implant} OR {corpus-based} OR laryngeal OR {speech-in-noise} OR {speaker identification} OR {language identification} OR computational OR {language acquisition} OR infant OR dyslexia OR {formant transition} OR {neural network} OR modelling ) ) ) AND (LIMIT-TO (DOCTYPE,"ar”))

**Web of Science (n= 463 Articles)**

TS=((Acoustic* OR “acoustic cue” OR “acoustic correlate” OR “audio cue” OR “voice stress” OR “acoustic analysis” OR pitch OR “fundamental frequency” OR “sound properties” OR psychoacoustic* OR prosod*) ) AND TS=((stress OR “cognitive load” OR anger OR frustrat* OR tension OR "negative affect" OR aggress* OR "negative emotion") ) AND TS=((speech OR "oral communication" OR "voice communication") ) NOT TS=((animal OR child* OR schizophrenia OR depress* OR autism OR dementia OR Alzheimer OR Huntington OR Parkinson`s disease OR aphasia OR dysphonia OR impairment OR damage OR disorder OR disabilities OR “machine learning” OR “lexical stress” OR “linguistic stress” OR “word stress” OR “simulated speech” OR music OR “compound stress” OR “glottal stop” OR singing OR trans OR “cochlear implant” OR “corpus-based” OR laryngeal OR “speech-in-noise” OR “speaker identification” OR “language identification” OR computational OR ”language acquisition” OR infant OR dyslexia OR “formant transition” OR “neural network” OR modelling) )

**PubMed (n=352 Articles)**

(Acoustic OR “acoustic cue” OR “acoustic correlate” OR “audio cue” OR “voice stress” OR “acoustic analysis” OR pitch OR “fundamental frequency” OR “sound properties” OR psychoacoustic OR prosodic) AND (stress OR “cognitive load” OR anger OR frustration OR tension OR "negative affect" OR aggression OR "negative emotion") AND (speech OR "oral communication" OR "voice communication") NOT (animals OR children OR schizophrenia OR depression OR autism OR dementia OR Alzheimer OR Huntington OR Parkinson`s disease OR aphasia OR dysphonia OR impairment OR damage OR disorder OR disabilities OR “machine learning” OR “lexical stress” OR “linguistic stress” OR “word stress” OR “simulated speech” OR music OR “compound stress” OR “glottal stop” OR singing OR trans OR “cochlear implant” OR “corpus-based” OR laryngeal OR “speech-in-noise” OR “speaker identification” OR “language identification” OR computational OR ”language acquisition” OR infant OR dyslexia OR “formant transition” OR “neural network” OR modelling)

**PsychInfo (n=330 articles if limited by peer-reviewed journals):**

(((Acoustic or "acoustic cue" or "acoustic correlate" or "audio cue" or "voice stress" or "acoustic analysis" or pitch or "fundamental frequency" or "sound properties" or psychoacoustic or prosodic) and (stress or "cognitive load" or anger or frustration or tension or "negative affect" or aggression or "negative emotion") and (speech or "oral communication" or "voice communication")) not (animals or children or schizophrenia or depression or autism or dementia or Alzheimer or Huntington or Parkinson`s disease or aphasia or dysphonia or impairment or damage or disorder or disabilities or "machine learning" or “lexical stress” or “linguistic stress” or “word stress” or “simulated speech” OR music OR “compound stress” OR “glottal stop” OR singing OR trans OR “cochlear implant” OR “corpus-based” OR laryngeal OR “speech-in-noise” OR “speaker identification” OR “language identification” OR computational OR ”language acquisition” OR infant OR dyslexia OR “formant transition” OR “neural network” OR modelling)).ab,hw,id,ot.

- **2102 records identified**
- After elimination of 671 duplicate records, **1431** records were **screened.**
