# Supplementary material for: Measuring negative emotions and stress through acoustic correlates in speech: A systematic review
Source: PLoS One. 2025 Jul 24;20(7):e0328833. doi: 10.1371/journal.pone.0328833 (PMC12289014; doi:10.1371/journal.pone.0328833)
Supplement: S2 Table — (DOCX) [file pone.0328833.s003.docx]

**S2 Table. Quality Assessment with the MMAT.**

***N = 37***

For every point in each sub question, the study gets 20% of quality criteria met. There are always 5-sub question per category.

For every 0.5 points in the sub questions, the study gets 10% of quality criteria met.

For every 0 points, no % will be given.

The overall quality criteria assessment is the addition of all percentages from each sub questions. Therefore, each study can have met between 0-100% of quality criteria. Please see the document “Threshold quality assessment” for the questions for each subcategory.

**Questions for quantitative randomized studies:**

1. Is randomization appropriately performed?
2. Are the groups comparable at baseline?
3. Are there complete outcome data? (Only include when possible, otherwise N/A)
4. Are outcome assessors blinded to the intervention provided?
5. Did the participants adhere to the assigned intervention?

**Questions for quantitative non-randomized studies:**

1. 1 Are participants representative of the target population?
2. Are measurements appropriate regarding both the outcome and intervention (or exposure)?
3. Are there complete outcome data?
4. Are the confounders accounted for in the design & analysis?
5. During the study period, is the intervention administered (or exposure occurred) as intended?

**Questions for qualitative studies:**

1. Is the qualitative approach appropriate to answer the research question?
2. Are the qualitative data collection methods adequate to address the research question?
3. 3 Are the findings adequately derived from the data?
4. Is the interpretation of results sufficiently substantiated by data?
5. Is there coherence between qualitative data sources, collection, analysis and interpretation?

| **Study ID** | **Author(s), publication year** | **Origin of study** | **Study Design** | **Emotion or stress being measured** | **Acoustic Correlates** | **Results** | **Quality Assessment MMAT**  (20% per question that is answered with yes) |
| --- | --- | --- | --- | --- | --- | --- | --- |
| 1 | Abur et al. (2023) | Boston, USA | Experiment | Cognitive Load | CPP L/H ratio  F0  Sound pressure level | No measures statistically significant | **Quantitatively non-randomized study**  1) Clear description of population. Inclusion and exclusion criteria given. No information on how participants were recruited (we don’t know if participants are representative of target population). **🡪 10%**  2) Yes; Measurement of Cognitive Load with the Stroop Task. Acoustic Analysis with the PRAAT-Software. **🡪 20%**  3) No dropouts mentioned. **🡪 20%**  4) Only healthy participants without diagnoses and psychological disorders were included. No other confounding variables like smoking & alcohol were considered **🡪 10%**  5) yes; 3 physiologic measures of autonomic arousal changed with CL 🡪 **20%**  **🡪 70% quality criteria met** |
| 2 | Alvear et al. (2012) | Malaga, Spain | Experiment | Stress | F0 | F0 increases | **Quantitatively non-randomized study**  1) Clear description of population and inclusion criteria. No description on how and where participants were recruited. **🡪 10%**  2) mental arithmetic task in order to induce mental stress; Multi-Dimensional Voice Program (MDVP) Model 5105, KayPENTAX™ for speech analysis **🡪 20%**  3) No dropouts 🡪 **20%**  4) Only healthy participants; no one was under medication. But no control for other confounders 🡪 **10%**  5) An increase in heart rate was observed, which is an indicator that stress levels of the participants indeed increased as a result of the intervention 🡪 **20%**  **🡪 80% quality criteria met** |
| 3 | Biassoni et al. (2016) | Milan, Italy | Experiment | Hot Anger, Cold Anger | F0 (mean, std, min, max)  Energy (mean, std, min, max)  Time (vocal string length, speech length, number and duration of pauses) | Increase in F0 min range  Increase in max Energy  No other changes in F0 and energy  No changes in time parameters | **Quantitatively Randomized Study**  1) No information on how participants were randomly assigned to each condition (reappraisal condition vs. control condition).. No information on allocation process given (double-blinded experimenter?) **🡪 0%**  2) Yes, groups are comparable as balanced group sizes were given as well as an A 2x2- design. **🡪 20%**  3) Dropout of 3 participants due to mistakes following the experimenter`s instructions (data was excluded); this makes 7% of data missing, which is not over the threshold **🡪 20%**  4) no information given, if outcome assessors were aware of who is receiving the intervention **🡪 10%**  5) Yes; adherence was optimal; participants remained in their condition. **🡪 20%**  **🡪 70% quality criteria met** |
| 4 | Bonner (1943) | South Carolina, USA | Experiment | Fear/Tension | F0, range of F0  Rhythm (Hypha-time, pause-time and total time) | **No constant trend.**  Increased F0  Wider F0 range  Hypha (syllable)-length increase  Longer pause-time | **Quantitatively non-randomized study**  1) participants are clearly described but no mention of inclusion & exclusion criteria or recruitment process **🡪 0%**  2) Inexperienced students were brought in front of a microphone to present sth. No information given on how the speech analysis was conducted**🡪 10%**  3) Yes, complete data. **20%**  4) no, no information of confounders accounted for **🡪 0%**  5) Self-report on the degree of emotional tension in the present situation to determine presence of affective states **🡪 20%**  **🡪 50% quality criteria met** |
| 5 | Boyer et al. (2018) | Toulouse, France | Experiment | Cognitive Load | Mean F0, Fmod,  Jitters (J1, J2, RAP, PPQ5, DDP)  Shimmers (S1, S2, APQ3, APQ5, APQ11, DDA)  N, DAL, DALT0, DALT0/T0, jittDALT0, jittDALT0/T0  Mean HNR, SDHNR  F1, F2, F3, F4  Spectral center of gravity  Normalized skewness  Normalized kurtosis  Twelve MFCCs  Three energy differences between two bands of the vowel`s fast Fourier transform spectrum  EBF (in HZ)  Spectral tilt (db/Hz) | F0 and SDF0 increase significantly  Shimmer 1 and FMod decrease significantly  No effect on other shimmers  N increases significantly  DALT0 decreases significantly  No significant changes in DALT0/T0  JittDal0/T0 and jitDal/T0 decrease significantly  HNR varies significantly  No other spectral parameters vary significantly | **Quantitatively non-randomized study**  1) Clear description of sample (students). Inclusion criteria described. Students took part on a voluntary basis, however no information on the recruitment process is provided. 🡪 **10%**  2) Yes; Memory task (Recall) appropriate for inducing mental load. PRAAT Software for speech analysis. 🡪 **20%**  3) Yes; no dropouts 🡪 **20%**  4) Yes, control for drinking caffeine or alcohol at least 6 hours before experiment; no smokers. **🡪 20%**  5) Yes; pupil size was used and varied significantly between most of the seven mental load conditions **🡪 20%**  **🡪 90% quality criteria met** |
| 6 | Brenner et al. (1994) | Washington DC, USA | Experiment | Cognitive Load | F0  Speaking rate (arts per second)  Vocal intensity (loudness)  Vocal jitter  Vocal shimmer  Derived speech measure (combines properties of several speech measures) | F0 increases  Increase in loudness (intensity)  Increase in speaking rate  No effect on vocal jitter and vocal shimmer  Derived speech measure increases significantly, especially when jitter was excluded (higher strength) | **Quantitatively non-randomized study**  1) Clear description of sample (only males), no exclusion or inclusion criteria mentioned. No information on where and how study participants were recruited. **🡪 0%**  2) Yes; Manual tracking task (counting task) to induce workload. For speech analysis, a speech analysis program was developed (reliability test or validity missing) **🡪 10%**  3) Yes. no dropouts 🡪 **20%**  4) No. no confounders were accounted for. **🡪 0%**  5) Yes. Heart rate data was collected to assess if stress was indeed successfully induced. **🡪 20%**  🡪 **50% quality criteria met** |
| 7 | Bucharan et al. (2014) | Missouri, USA | Experiment | Stress | Total number of WPM  Number of pauses  Duration of pauses | Number of pauses and duration of pauses increase  Total number of WPM not associated with physiological indices of stress | **Quantitatively non-randomized study**  1) Clear description of sample. Clear information on Inclusion and Exclusion criteria but no information on recruitment process and study participants. **🡪 10%**  2) Yes. Trier Social Test to induce stress. Calculations for acoustic measures appropriate **🡪 20%**  3) Yes, no dropouts **🡪 20%.**  4) Confounders like menstrual cycle and oral contraceptive use were assessed. No other confounders were assessed (e.g. smoking, drinking caffeine) **🡪 10%**  5) Yes. Saliva and Heart rate measures were analyzed to assess if stress induction was successful. **🡪 20%**  **🡪 80% quality criteria met.** |
| 8 | Bulling et al. (2020) | Zurich, Switzerland | Experiment | Stress | F0 | F0 increases | **Quantitatively non-randomized study**  1) Clear description of sample. No information on exclusion or inclusion criteria or recruitment process. **🡪 10%**  2) Yes. Trier Social Test induces stress. PRAAT is appropriate for F0 analyses. 🡪 **20%**  3) Yes, no dropouts **🡪 20%**  4) No confounders were assessed 🡪 **0%**  5) Yes. The topic of conversation and the type of stress expression was coded to assess stress states. 🡪 **20%**  **🡪 70% quality criteria met.** |
| 9 | Congleton et al. (1997) | Texas, USA | Experiment | Cognitive Load | F0  Jitter  Shimmer | F0 increases significantly  Less consistent inverse relationship between jitter and cognitive load  No changes in shimmer. | **Quantitatively non-randomized study**  1) Description of sample clear. Not enough information given regarding inclusion and exclusion criteria. No information on recruitment of participants provided, except that they had volunteered to participate in the study **🡪 0%**  2) Yes. Simulation of Airborne Warning system to simulate stress at work. Pitch extraction program SWIFFT was used. **🡪 20%**  3) Yes, no dropouts known. **🡪 20%**  4) No confounders have been accounted for besides pregnancy. **🡪 0%**  5) No, no additional analyses. Two independent measures of stress have been employed to identify times of high workload but no further information given **🡪 10%**  **🡪 50 % quality criteria met** |
| 10 | Fuller et al. (1992) | Colorado, USA | Field Study | Anxiety | Mean F0  Jitter  Tenseness | Lack of validity for mean F0 changes  F2 changes significantly, mixed results for other tenseness measures  Valid jitter changes | **Quantitatively non-randomized study**  1) Clear description of the sample (only women). Exclusion criteria stated; students recruited by campus advertisement. **🡪 20%**  2) No, subject’s exam for inducing anxiety is accurate but Accelerometer for jitter and shimmer is not recognized as a precise measurements. **🡪 10%**  3) Yes, no known dropouts **🡪 20%**  4) No confounders have been accounted for besides pregnancy for women. **🡪 0%**  5) Yes, physiological arousal measured based on heart rate, palmar sweating, and forehead muscle tension **🡪 20%**  **🡪 70% quality criteria met** |
| 11 | Griffin & Williams (1987) | Florida, USA | Experiment | Stress | F0  Intensity  Word duration in ms | F0 increases significantly  Intensity increases significantly  Word duration decreases significantly (rapid speech) | **Quantitatively Non-randomized Study**  1) No clear description of sample (e.g., gender and age are not known). No inclusion and exclusion criteria mentioned. No information on recruitment. Information on selection process of subset is provided.  **🡪 0%**  2) Yes, complex psychomotor and dichotic task represents work environment. Kay Elemetrics Model 6087 for speech analysis. **🡪 20%**  3) Yes; No dropouts **🡪 20%**  4) No confounders have been accounted for. **🡪 0%**  5) No other measurements were performed to measure stress.  **🡪 0%**  **🡪 40% quality criteria met** |
| 12 | Hall et al. (2021) | Wales, UK | Experiment (Simulation) | Stress | F0  F1, F2, F3, F4 | F0 changes significantly  No changes in F1,F2, F3, F4 | **Quantitatively non-randomized study**  1) No information on surgeons’ age or expertise given. No information on inclusion or exclusion criteria. Recruitment via simulation team training program **🡪 0%**  2) Yes, simulation is inducing stress. PRAAT is a software to analyze F0 and Formant Frequencies. **🡪 20%**  3) Yes, no dropouts mentioned. **🡪 20%**  4) No; only some confounders (e.g. gender) have been considered, but other potential confounders were not assessed (e.g. coffee intake, smoking etc.) **🡪 0%**  5) No additional measures**. 🡪 0%**  **🡪 40% quality criteria met** |
| 13 | Hecker et al. (1968) | Massachusetts, USA | Experiment | Stress | F0  F0 contour during an utterance  Amplitude of glottal pulses (level)  Shape or frequency spectrum of each pulse  Regularity in shape of successive pulses  Initiation of glottal vibration  Duration of phonetic segments  Precision of articulatory targets for vowels | No homogenous trend, increases and decreases in the acoustic correlates. | **Quantitative non-randomized study**  1) No information on participants given (age, gender). No information on inclusion and exclusion criteria and on the recruitment process **🡪 0%**  2) Yes. Arithmetic Task under time pressure causes stress. Narrow-band spectrograms were used to analyze F0. **🡪 20%**  3) No measurement of F0 could be obtained in some cases, as there was noise in the data and vibration of the vocal folds were irregular. (No mention in how many cases this was the case). In addition, some participants were insufficiently stressed during the experiment and some not fully relaxed during control condition; however, it was not clear for how many participants this was the case. **🡪 0%**  4) No confounders considered. **🡪 0%**  5) Yes. Movements, Respiratory activity and skin potential were considered.  **🡪 20%**  **🡪 40% quality criteria met.** |
| 14 | Hodgins et al. (2010) | New York, USA | Experiment | Threat Response | Mean F0 | F0 increases in perception of controllability  Less increased F0 and threat response in autonomous primed motivation. | **Quantitatively non-randomized study**  1) Clear descriptions of sample. No inclusion or exclusion criteria mentioned. Missing information on recruitment. **🡪 0%**  2) Structured stressful interview with moderately high social threat leads to threat response. Amadeus Software was used for speech analysis. **🡪 20%**  3) Yes. No dropout data mentioned. **🡪 20%**  4) Experimenter effect was controlled for as a potential confounding variable.. But no control for confounders in voice signal. **🡪 10%**  5) Yes. Physiological markers (cardiovascular responses) measured for correlation of verbal and physiological cues **🡪 20%**  **🡪 70% quality criteria met.** |
| 15 | Huttunen et al. (2011a) | Oulo, Finland | Experiment | Cognitive Load | Articulation rate  Mean F1 and F2 front vowel  Mean F1 and F2 back vowel | Articulation rate decreases significantly  Mean F1 and F2 back vowel increase significantly  Increase in mean F1 front vowel, decrease in mean F2 front vowel | **Quantitatively non-randomized study**  1) Clear description of the sample (only males). No inclusion or exclusion criteria mentioned. No information on recruitment process provided **🡪 0%**  2) Yes, Simulated Military Flight inducing cognitive Load. Algorithm and PRAAT used to extract features. **🡪 20%**  3) Yes, outcome data complete. **🡪 20%**  4) No confounders were accounted for. **🡪 0%**  5) Yes. Subjective assessment by the flight instructor using the visual analogue scale (VAS). **🡪 20%**  **🡪 60% quality criteria met.** |
| 16 | Huttunen et al. (2011b) | Oulo, Finland | Experiment | Cognitive Load | Mean F0 utterance-level  Mean vocal Intensity | F0 increases  Mean vocal intensity increases | **Quantitative non-randomized study**  1) Clear description of the sample (male flight pilots). No information on the recruitment process. No exclusion/inclusion criteria given**. 🡪 0%**  2) Yes, simulator flight represents the cognitive load in this context. Speech Analysis performed with PRAAT. **🡪 20%**  3) No, outcome data from 2 pilots. Under the threshold of 20%, with 13.3% data missing. missing due to unexpected failure in audio or video recordings. **🡪 20%**  4) Yes, living habits as confounding factors have been assessed (e.g. smoking, coffee) **🡪 20%**  5) Yes. Subjective assessment by the flight instructor. **🡪 20%**  **🡪 80% quality criteria met.** |
| 17 | Kandsberger et al. (2016) | St. Andrews, UK | Experiment | Emotional Stress | Mean F0 | Mean F0 increases | **Quantitative non-randomized study**  1) Yes. inclusion and exclusion criteria were defined. Recruitment process and participant information were provided**. 🡪 20%**  2) Yes. PRAAT Software for Speech Analysis and Stress measurement in follow-up consultations were used. **🡪 20%**  3) No outcome data missing. **🡪 20%**  4) No confounding variables were assessed in this study. **🡪 0%**  5) Yes. Verona Coding Definitions of Emotional Sequences (VR-CoDES) were compared to F0 measures **🡪 20%**  **🡪 80% quality criteria met.** |
| 18 | Kappen et al. (2022) | Ghent, Belgium | Experiment | Psychosocial Stress | F0  HNR  Shimmer  Jitter  Speech rate | F0 increases  HNR increases significantly  Shimmer decreases  No effect in jitter  No effect on speech rate | **Quantitative non-randomized study**  1) Yes. Participant and recruitment process information given. Inclusion criteria mentioned in supplementary material. **🡪 20%**  2) Yes. Psychosocial stress induction task with negative evaluation were used to induce stress. OpenSmile was used for speech analysis. **🡪 20%**  3) From 77 participants, only audio recordings of 71 participants could be analyzed due to audio/recording quality. Under threshold **🡪 20%**  4) No confounding variables have been accounted for besides gender. **🡪 0%**  5) Physiological measures (heart rate acceleration) and self-report to validate stress **🡪 20%.**  **🡪 80% quality criteria met.** |
| 19 | Kappen et al. (2024) | Ghent, Belgium | Experiment | Acute (psychosocial) Stress | F0  Jitter  Shimmer  HNR  Speech rate  Voiced segment length | **MIST stress paradigm:** - significant increase in F0 - significant increase in voiced segments per second - significant increase in voiced segment length  - significant decrease in Jitter **Cyberball:** - no significant increases/changes  no significant change in HNR and shimmer.  Cyberball did not elicit physiological and self-reported stress response. | **Quantitative non-randomized study**  1) Yes. Recruitment process through social media, participant info given. Exclusion criteria were described in supplementary material. **🡪 20%**  2) Yes. MIST & Cyberball measure different kind of stress. Cyberball measures social stress while MIST measures cognitive stress. OpenSmile was used for speech analysis. **🡪 20%**  3) Yes. 8 of the control group recordings were removed (from 128) because of quality issues. Still, 66 participant’s recordings could be included. Under threshold. **🡪 20%**  4) No confounding variables have been accounted for besides gender. **🡪 0%**  5) Yes. Skin conductance response rate (SCRR) & self-reports. No increase in skin conductance & self-reported stress during Cyberball. **🡪 20%**  **🡪 80% quality criteria met.** |
| 20 | Lebedeva & Shved (2022) | Moscow, Russia | Experiment | Anxiety | F0  Intensity  Number of vocal impulses  Pause duration (unvoiced segments)  Jitter  Shimmer | Decrease in intensity  Longer speech Pauses  Increased shimmer  no other measures reported. | **Quantitative non-randomized study**  1) Sample description given (4 men, 2 women). No information on inclusion/exclusion criteria and no information on how recruitment was being carried out **🡪 0%**  2) 14-day isolation and crowding during a space simulation may induce anxiety. Speech Analysis with PRAAT **🡪 20%**  3) Some acoustic correlates have been evaluated but it seems that all non-significant results are not described further. We don`t know what is with this missing values **🡪 0%**  4) No confounding variables have been looked for 🡪 0%  5) STAI was used to assess anxiety levels and link them to speech data **🡪 20%**  **🡪 40% quality criteria met** |
| 21 | Lee & Redford (2015) | Oregon, USA | Experiment | Cognitive Load | F0 range  Normalized measure of F0 variation  Mean normalized sequential variability in vowel durations (nPVI)  Error rates  Prosodic breaks  Articulation rate | Error rate increases  Faster speech  Fewer prosodic breaks  No effect on spoken rhythm & nPVI  No effect on F0 correlates | **Quantitative non-randomized study**  1) Sample description given (7 female out of 20). Inclusion/Exclusion criteria given. Recruitment from a student pool, no information on how recruitment process was carried out **🡪 10%**  2) Yes. Span task to induce cognitive load. Speech Analysis with PRAAT. **🡪 20%**  3) Yes. Only one dropout of ones participants’ data because of technical problems. Under threshold. **🡪 20%**  4) No confounding variables have been accounted for. **🡪 0%**  5) No other measurements taken to validate cognitive load. **🡪 0%**  **🡪 50% quality criteria met.** |
| 22 | Li et al. (2023) | Chengdu, China | Experiment | Anxiety | F0 mean  Short-term energy (STE)  Formant Frequency (FM/F1)  MFCC1-MFCC12  Brightness (power in speech signals) | F0 mean  MFCC1std  FMstd  STEstd  All correlated with self-reported anxiety | **Quantitative non-randomized study**  1) Description of sample given. No Inclusion and exclusion criteria given. No information on how students were recruited**🡪 0%**  2) Speech/presentation for inducing anxiety. Information on how features were extracted not clear. **🡪 10%**  3) Yes. No dropouts mentioned. **🡪 20%**  4) Relevant psychological traits were taken into account. But no other confounding variables were assessed. **🡪 10%**  5) Yes. Behavioral signals as indices of anxiety state (gaze, facial expressions, body movements, gestures, postures), speakers’ self-perceived anxiety levels & human raters **🡪 20%**  **🡪 60% quality criteria met.** |
| 23 | Lively et al. (1993) | Indiana, USA | Experiment | Cognitive Load | Amplitude  Amplitude variability  Spectral tilt  F0  F0Std  Duration (phrase duration)  Formant Frequencies | Increased amplitude  Sign. Increase in amplitude variability from one utterance to the next in 4 out of 5 speakers.  Spectral tilt decreases under, without associated change in amplitude.  No consistent effect on F0  Significant decrease in F0 Std.  Significant shorter phrase duration  No significant change in Formant Frequencies | **Quantitatively non-randomized study**  1) No clear description of the sample (age missing) and of the recruitment process. Inclusion criteria given. **🡪 0%**  2) Visual tracking task to induce cognitive load. Speech analysis with digital signal processing techniques and linear predictive coding to calculate short-term-spectrum **🡪 20%**  3) No missing outcome data **🡪 20%**  4) No confounding variables have been assessed**. 🡪 0%**  5) No additional measures **🡪 0%**  **🡪 40% quality criteria met.** |
| 24 | MacPherson et al. (2017) | Boston, USA | Experiment | Cognitive Load | F0  Sound pressure level  cepstral peak prominence (CPP)  L/H ratio | Increased CPP  Lower L/H ratio  No change in F0 or sound pressure level | **Quantitative non-randomized study**  1) Clear description of the sample. Inclusion criteria given. No information on recruitment given. **🡪 10%**  2) Yes. Stroop Task induces cognitive load. MATLAB used for speech analysis. **🡪 20%**  3) No dropouts mentioned. **🡪20%**  4) Yes. Confounding factors have been accounted for (e.g. smoking, health  conditions affecting autonomous reactions) **🡪 20%**  5) Yes. Autonomic measures (pulse & skin conductance) **🡪 20%**  **🡪 90% quality criteria met.** |
| 25 | Mendoza & Carballo (1998) | Granada, Spain | Experiment | Cognitive Load | F0, F0 range, F0 (STD)  Jitter  Shimmer  Noise-to-Harmonic Ratio (NHR)  High-frequency harmonic energy (SPI)  Spectral noise (VTI) | F0 increases  Decreased jitter  Decreased shimmer  Increased SPI  Decreased VTI  No other changes.  Effect still visible after stressful demand in performance is being removed (except in SPI) | **Quantitative non-randomized study**  1) No information on sex distribution, only that both sexes are included. Inclusion criteria given. Participants are students enrolled in a course. **🡪 10%**  2) Yes, cognitive tasks induce cognitive load. MDVP is used for speech analysis. **🡪 20%**  3) Yes. No dropouts mentioned. Outcome complete. **🡪 20%**  4) No confounding variables have been accounted for. **🡪 0%**  5) Yes. Same experiment was conducted without stressful demand in performance in order to validate, that experimental task really induced workload. This is a control condition but no other variables were assessed to validate the stress measured. **🡪 0%**  **🡪 50% quality criteria met.** |
| 26 | Pisanski & Sorokowski (2021) | Wroclaw, Poland | Field Study | Stress | F0, F0 mean, F0 min, F0 max, F0SD  F1-F4  Formant spacing (DeltaF)  HNR  Jitter  Shimmer  Speed of speech (duration and words per minute WPM) | F0 increased  Greater DeltaF  Increased speed of speech (more WPM, shorter duration)  No other changes. | **Quantitative non-randomized study**  1) Clear description of the sample (only females), registered in a psychology course. No information on recruitment or selection process for these students were provided. Inclusion and exclusion criteria provided **🡪 10%**  2) real-life oral examination situations induce stress. Speech Analysis with PRAAT. **🡪 20%**  3) Yes. No known dropout data. **🡪 20%**  4) No confounding variables were accounted for. **🡪 0%**  5) Cortisol measures (saliva sample) and baseline measure of voice were performed 2 weeks before the oral exam. There might be a bias in cortisol measure because it is not possible to compare measures taken two weeks apart. **🡪 10%**  **🡪 60% quality criteria met.** |
| 27 | Pisanski et al. (2016) | Wroclaw, Poland | Field Study | Stress | mean F0  F0 min  F0 max  F0SD | F0 mean increased significantly  F0 min increased significantly  No sign. Changes in F0SD and F0 max. | **Quantitative non-randomized study**  1) Clear description of the sample (only females), including participants registered in a psychology course. No information on the recruitment or selection process was provided. Inclusion and exclusion criteria given **🡪 10%**  2) real-life oral examination situation induces stress. Speech Analysis with PRAAT. **🡪 20%**  3) Dropout of 3 participants that did not meet the criteria after prescreening. Dropout under threshold **🡪 20%**  4) Yes, confounding variables have been accounted for (smoking, drinking etc.). **🡪 20%**  5) Cortisol measures (saliva sample) and baseline measure of voice were taken 2 weeks before oral exam. There might be a bias in cortisol measure because it is not possible to compare measures taken two weeks apart.**🡪 10%**  **🡪 80% quality criteria met.** |
| 28 | Pisanski et al. (2018) | Wroclaw, Poland | Experiment | Psychosocial Stress | F0 mean, F0 range, F0 variation  Jitter  Shimmer  HNR | F0 mean, F0 range and F0 variation change significantly  No other significant changes | **Quantitative non-randomized study**  1) Yes. Description of the recruitment process and of the participants provided (more women, n= 57) than men, n= 33). Inclusion and exclusion criteria given **🡪 20%**  2) Trier social stress test induces stress reaction. PRAAT for speech analysis. **🡪 20%**  3) 8 participants dropped out after the second screening process. Still under the threshold. **🡪 20%**  4) Confounding factors have been considered in the second screening process. Participants were told not to smoke, drink etc. before. **🡪 20%**  5) Baseline comparison of cortisol was taken 2 weeks before. Not the best study design. **🡪 10%**  **🡪 90% quality criteria met.** |
| 29 | Rochman et al. (2008) | Beer Sheva, Israel | Experiment | Unresolved Anger | Mean F0  F0 range  Amplitude range  F0 perturbation (PPQ)  Amplitude perturbation (APQ)  Articulation rate (WPM) | Mean F0 increased significantly  F0 range increased significantly  WPM increases significantly  Amplitude range increases significantly  No increase in APQ and PPQ | **Quantitative non-randomized study**  1) Clear description of sample (female university students). Information on recruitment process and Inclusion and Exclusion criteria given **🡪 20%**  2) Yes. Mood induction task to induce anger. PRAAT for speech analysis. 🡪 **20%**  3) One data of a person missing because he did not express anger. Under threshold. **🡪 20%**  4) No, no confounding variables have been accounted for. **🡪 0%**  5) Yes. Self-reports to validate the results of speech analysis. **🡪 20%**  🡪 **80% quality criteria met.** |
| 30 | Ruiz et al. (1996) | Toulouse, France | 1) Experiment 2) Field Study | Stress | Mean F0  µ index  Δ area  Frequency distribution of sound level (CSPD)  Spectral balance frequency Fs  F1, F2, F3  Distances to the F1-F2-F3 space center | 1) F0 increases significantly  µ index increases significantly  significant variations in F1,F2,F3  No relation with Δ area variations  No effect on F1-F2-F3 space center  2) mean F0 increases significantly | **Study 1: Quantitative non-randomized study**  1) One young subject, not enough clear information on this person given. No information about inclusion or exclusion criteria provided. No information on recruitment **🡪 0%**  2) Stroop task induces cognitive load and not stress. Speech Analysis with tracking algorithm implemented on 5500 Kay analyzers & CSL 4300 **🡪10%**  3) No missing outcome data. **🡪 20%**  4) No confounding variables were considered. **🡪 0%**  5) No additional measures. **🡪 0%**  **🡪 30% quality criteria met.**  **Study 2: Qualitative study – Case Study**  1) Yes, the qualitative approach is appropriate, because stress in real life situation was investigated**. 🡪 20%**  2) Yes, data collection method is adequate, as audio data of both the pilot and copilot were analyzed **🡪 20%**  3) Yes, findings are adequately derived from the data. **🡪 20%**  4) Interpretation of the results is supported by the data collected. Nevertheless, due to the study design, we cannot be 100% sure of the classification of no stress/stress in the voice signal, as no other measures could be taken as a control variable **🡪 10%**  5) There is a clear link between data collection, analysis and interpretation **🡪 20%**  **🡪 90% quality criteria met** |
| 31 | Sabo & Rajcani (2017) | Bratislava, Slovakia | Experiment | Stress | Mean F0  Mean Intensity | Mean F0 increases significantly in 4 out of 5 speakers  Speech intensity increases | **Quantitative non-randomized study**  1) Clear description of the sample. No information provided on where the recruitment took place (online/on site) and bias of participation. No inclusion or exclusion criteria due to its preliminary study nature **🡪 0%**  2) This computer task “dismantling a bomb” is known to cause stress. No information on how speech was analyzed **🡪 10%**  3) No outcome data missing. **🡪 20%**  4) No confounding variables have been accounted for **🡪 0%**  5) Physiological data (heart rate) were collected **🡪 20%**  **🡪 50% quality criteria met** |
| 32 | Sobin & Alpert (1999) | New York, USA | Experiment | Hate, Anger | F0, F0 variance  Volume, volume variance  Speaking rate  Duration of speech  Duration of pauses  Number of pauses | Fear:  Increased F0, F0 variance, volume, speaking rate  Decreased speech duration, duration of pauses, number of pauses  Anger: Increased F0 variance, volume, volume variance,  Decreased pitch, speaking rate, speech duration, duration of pauses and number of pauses | **Quantitative non-randomized study**  1) Description of the sample sufficient. Exclusion criteria (psychiatric disorder) stated only indirectly because data of two participants was excluded because of psychiatric symptoms. No information on recruitment process **🡪 10%**  2) We don’t know, if emotion induction procedure by reading emotion-induction stories really lead to these emotions. Speech analysis was done by human raters and “utterance detail program” focusing on prosodic features. **🡪 10%**  3) Out of 35 participants, the data of 31 participants could be analyzed. Two did not complete the protocol and two subjects had positive psychiatric symptoms, therefore their data was excluded. Dropout of <20%, therefore below threshold **🡪 20%**  4) No confounders looked at. **🡪 0%**  5) Human ratings **🡪 20%**  **🡪 60% quality criteria met.** |
| 33 | Sondhi et al. (2015) | Haryana, India | Field Study | Stress | Mean F0  F1, F2, F3, F4 | Mean F0 increases  F1, F2, F3, F4 decrease | Qualitative study – Field Study  1) Qualitative approach appropriate, as they wanted to look for stress in real-life situations **🡪 20%**  2) Data collection method (audio from participants) adequate. **🡪 20%**  3) Findings are adequately derived from the data. **🡪 20%**  4) interpretation of the results is not 100% supported by data, as pitch fall/rise is interpreted into certain emotional states such as embarrassment, neutral and angry (potentially over interpretation) 🡪 **0%**  5) not clear line between data collection, analysis and interpretation, as interpretation of subjective feelings seems a bit subjective **🡪 10%**  **🡪 70% quality criteria met.** |
| 34 | Streeter et al. (1983) | New Jersey, USA | Field Study | Stress | Mean F0, F0 max, F0 min, F0std,  Mean Amplitude, SD Amplitude, Max Amplitude  Number of words/s | No trend observable in speech analysis  Listeners perceive increased F0 amplitude levels and increased variability in F0 and amplitude levels as stressful | Qualitative study – Case study  1) Qualitative approach appropriate, because they wanted to investigate stress in real-life situations **🡪 20%**  2) Data collection method (tape recordings from SO & CSO) adequate 🡪 **20%**  3) Findings are adequately derived from the data (Linear predictive coding analysis & human listener ratings) 🡪 **20%**  4) Interpretation of results supported by the data **🡪 20%**  5) Clear line between data collection, analysis and interpretation. Discussion of potential influences of other factors was adequate **🡪 20%**  **🡪 100% quality criteria met** |
| 35 | Tavi (2017) | Joensuu, Finland | Field Study | Stress | Median F0  Jitter  Shimmer  Harmonics to noise ratio (HNR)  Hammarberg Index  Duration of /i/-vowels  F1, F2, F3  Dispersion between F1-F2 | higher F0  changes in shimmer  no changes in jitter  lower HNR  Lower Hammarberg index  No difference in duration of /i/-vowels under stress  Higher F1  Lower F2 and F3  Narrower Formant dispersion | Qualitative study – Field study  1) Qualitative approach appropriate, because they wanted to investigate stress in real-life situations **🡪 20%**  2) Data collection method (calls to emergency) adequate 🡪 **20%**  3) Findings are adequately derived from the data (PRAAT & ProsodyPro) 🡪 **20%**  4) Interpretation of the results not fully supported by the data. They categorized 5 recordings belonging to stress group and 8 recordings belonging to neutral group – but how can we be 100% sure the “non-stress” group is not feeling stressed? **🡪 10%**  5) Clear line between data collection, analysis and interpretation. **🡪 20%**  **🡪 90% quality criteria met** |
| 36 | Taylor et al. (2016) | Indiana, USA | Experiment | Social Stress | F0Std | greater deviation of F0std with F0 in social stress (significant) lower  greater deviation of F0std with F0 in problem-solving task higher (marginally significant) | **Quantitative non-randomized study**  1) 35 university students (17 women, 18 men); Recruitment process described (university students getting financial compensation); inclusion and exclusion criteria provided; nonetheless, the sample was extracted from a larger sample and is a convenience sample **🡪 20%**  2) Yes, experiment was designed to induce social stress. Analysis of speech data with sociometric badges**. 🡪 20%**  3) Only 9 of the 35 university students exhibited a cortisol response and therefore speech and cortisol data could only be correlated for these participants **🡪 0%**  4) No. Not enough confounding variables were considered. They only made sure that participants had not exercised before **🡪 0%**  5) Yes. Cortisol response was taken. 🡪 **20%**  **🡪 60% quality criteria met.** |
| 37 | Tolkmitt & Scherer (1986) | Giessen, Germany | Experiment | 1) Cognitive Load 2) Emotional Stress | F0  F1, F2  power spectra/ spectral energy | no significant change in F0 F0 floor increases vowel formants and power spectra changes ( Narrower formant bandwidth more precise spectral energy peaks  --> better articulation under high cognitive stress, but not under high emotional stress) | **Quantitative non-randomized study**  1) Mean age not described; not enough information on recruitment process. Out of 374 students, 33 males and 27 females were selected but no information about the selection process is provided. Exclusion and inclusion criteria not provided **🡪 0%**  2) Slide presentations showing 1) logical problems or 2) human bodies with diseases or injuries – manipulation check was carried out to make sure participants were really stressed. Analogue extraction with exact description how extraction was being performed. 🡪 **20%**  3) No missing outcome data. 🡪 **20%**  4) Confounding variables have not been assessed besides sex differences and anxiety deniers/high/low anxious. This might be because the study design was influenced by another research question, which did not required assessment of additional variables. **🡪 0%**  5) Subjective questionnaires (as manipulation check) to ensure they were really stressed. 🡪 **20%**  **🡪 60% quality criteria met.** |
| 38 | Wittels et al. (2002) | Vienna, Austria | Field Study | (Psychoemotional) Stress | F0 mode (F0 occurring most often) | F0 mode increased significantly | **Quantitative non-randomized study**  1) 30 male soldiers, enrolled in a special forces training – Clear description of the sample. Recruitment through the military course. No inclusion and exclusion criteria provided. **🡪 10%**  2) Yes. Real life condition of military training task induces stress for soldiers. Speech analysis with an algorithm developed by Lüdge & Gips **🡪 20%**  3) 4 soldiers’ biomedical data were incomplete and thus excluded from data analysis. Under threshold 🡪 **20%**  4) No confounding variables were assessed. **🡪 0%**  5) beat-by-beat electrocardiogram (ECG) measurement **🡪 20%**  **🡪 70% quality criteria met.** |
