# Supplementary material for: Measuring negative emotions and stress through acoustic correlates in speech: A systematic review
Source: PLoS One. 2025 Jul 24;20(7):e0328833. doi: 10.1371/journal.pone.0328833 (PMC12289014; doi:10.1371/journal.pone.0328833)
Supplement: S3 Table — (DOCX) [file pone.0328833.s004.docx]

# **S3. Characteristics of the studies included in the systematic review**

***N = 38***

| **Study ID** | **Author(s), publication year** | **Origin of study** | **Study Design** | **Sample Size** | **Age (years)** | **Male/Female** | **Other measures taken (physiological and subjective)** |
| --- | --- | --- | --- | --- | --- | --- | --- |
| 1 | Abur et al. (2023) | Boston, USA | Experiment | 12 older adults | 68-78 | 6 females, 6 males | Pulse volume amplitude, pulse period, skin conductance response amplitude. |
| 2 | Alvear et al. (2012) | Malaga, Spain | Experiment | 14 adults | n.i. | 7 females, 7 males | HR, systolic pressure (SP), diastolic pressure (DP), mean blood pressure (MP). |
| 3 | Biassoni et al. (2016) | Milan, Italy | Experiment | 44 adults | 20-28 | n.i. | No. |
| 4 | Bonner (1943) | South Carolina, USA | Experiment | 52 students | n.i. | 24 females, 28 males | Self-reports. |
| 5 | Boyer et al. (2018) | Toulouse, France | Experiment | 32 students | n.i. | 17 females, 15 males | Evoked pupillary response. |
| 6 | Brenner et al. (1994) | Washington DC, USA | Experiment | 17 adults | n.i. | 0 females, 17 males | Heart rate. |
| 7 | Bucharan et al. (2014) | Missouri, USA | Experiment | 91 healthy students | 18 – 25. 19.69 (mean)  SD= 2.4 | 51 females, 40 males. | Saliva samples  Heart rate. |
| 8 | Bulling et al. (2020) | Zurich, Switzerland | Experiment | 128 couples | Females = 26.4 mean (SD = 5.7) and males= 28.5 mean (SD = 6.3) | 64 females, 64 males. | Type of stress expression (coded by experts)  Topic of conversation (coded by experts). |
| 9 | Congleton et al. (1997) | Texas, USA | Experiment | 16 Air force Officers | 26 (mean) | 3 females, 13 males | Heart rate  Palmar sweating  Forehead muscle tension. |
| 10 | Fuller et al. (1992) | Colorado, USA | Field Study | 88 students | n.i. | n.i. | No. |
| 11 | Griffin & Williams (1987) | Florida, USA | Experiment | 20 student naval aviators (19 student pilots, 1 naval flight officer) | n.i. | n.i. | No. |
| 12 | Hall et al. (2021) | Wales, UK | Experiment (Simulation) | Surgeons | n.i. | n.i. | No. |
| 13 | Hecker et al. (1968) | Massachusetts, USA | Experiment | 10 (3 Air Force Pilots, 7 male employees) | n.i. | 0 female, 10 male | No. |
| 14 | Hodgins et al. (2010) | New York, USA | Experiment | 77 students | 18.6 (mean) | 47 female, 30 male | No. |
| 15 | Huttunen et al. (2011a) | Oulo, Finland | Experiment | 13 military pilots | 28 (mean) | 0 female, 13 male | No. |
| 16 | Huttunen et al. (2011b) | Oulo, Finland | Experiment | 13 military fighter pilots | 28 (mean) | 0 female, 13 male | No. |
| 17 | Kandsberger et al. (2016) | St. Andrews, UK | Experiment | 16 head and neck cancer survivors | 62.75 (mean) | 6 female, 10 male | No. |
| 18 | Kappen et al. (2022) | Ghent, Belgium | Experiment | 77 adults | 23.13 (mean),  SD = 6.19 | 50 females, 27 males | Self-reports  Heart rate acceleration. |
| 19 | Kappen et al. (2024) | Ghent, Belgium | Experiment | 66 adults | 21.29 (mean),  SD= 2.82 | 13 female, 53 male | Self-reports  Skin conductance response rate. |
| 20 | Lebedeva & Shved (2022) | Moscow, Russia | Experiment | 6 adults | 23-45 years | 2 female, 4 male | Self-reports. |
| 21 | Lee & Redford (2015) | Oregon, USA | Experiment | 19 students | n.i. | 7 female, 12 male | No. |
| 22 | Li et al. (2023) | Chengdu, China | Experiment | 75 students | n.i. | n.i. | Behavioral signals (gaze, facial expressions, body movements, gestures, postures)  Self-perceived anxiety levels  Human raters. |
| 23 | Lively et al. (1993) | Indiana, USA | Experiment | 5 male adults (3 psychology graduate students and 2 members of the laboratory staff) | n.i. | 5 male, 0 female | No. |
| 24 | MacPherson et al. (2017) | Boston, USA | Experiment | 16 young adults | n.i. | 8 female, 8 male | Pulse  Skin conductance. |
| 25 | Mendoza & Carballo (1998) | Granada, Spain | Experiment | 82 students | 18-22 |  | No. |
| 26 | Pisanski & Sorokowski (2021) | Wroclaw, Poland | Field Study | 10 students | n.i. | 10 female, 0 male | Cortisol (saliva). |
| 27 | Pisanski et al. (2016) | Wroclaw, Poland | Field Study | 34 students | 21-32 | 34 female, 0 male | Cortisol (saliva). |
| 278 | Pisanski et al. (2018) | Wroclaw, Poland | Experiment | 80 adults | n.i. | 47 female, 33 male | Biofeedback Polygraph with finger sensors (tracks changes in pulse, skin temperature, skin conductance, hand movement)  Cortisol (saliva). |
| 29 | Rochman et al. (2008) | Beer Sheva, Israel | Experiment | 17 students | 22-24 | 17 female, 0 male | Self-reports  Behavioral signals (SPAFF: specific affect coding system). |
| 30 | Ruiz et al. (1996) | Toulouse, France | 1) Experiment 2) Field Study | 1) 1 young adult  2) 1 Pilot & 1 Copilot | n.i. | n.i. | No. |
| 31 | Sabo & Rajcani (2017) | Bratislava, Slovakia | Experiment | 5 adults | n.i. | 2 female, 3 male | Beat-to-beat heart rate signal. |
| 32 | Sobin & Alpert (1999) | New York, USA | Experiment | 31 adults | n.i. | 31 female, 0 male | No. |
| 33 | Sondhi et al. (2015) | Haryana, India | Field Study | n.i. | n.i. | 0 female, 2 male | No. |
| 34 | Streeter et al. (1983) | New Jersey, USA | Field Study | 2 adults | n.i. | 2 male | Ratings of stress by two controllers  Final report outlining stressful events. |
| 35 | Tavi (2017) | Joensuu, Finland | Field Study | 13 adults | n.i. | 13 female | No. |
| 36 | Taylor et al. (2016) | Indiana, USA | Experiment | 35 students | n.i. | 17 female, 18 male | Cortisol (saliva). |
| 37 | Tolkmitt & Scherer (1986) | Giessen, Germany | Experiment | 60 students | n.i. | 27 female, 33 male | Questionnaire on subjective feelings. |
| 38 | Wittels et al. (2002) | Vienna, Austria | Field Study | 26 soldiers | n.i. | 0 female, 26 male | Heart rate. |
